# Functional characterization of the upstream components of the Hog1-like kinase cascade in hyperosmotic and carbon sensing in *Trichoderma reesei*

**DOI:** 10.1186/s13068-018-1098-8

**Published:** 2018-04-04

**Authors:** Zhixing Wang, Ning An, Wenqiang Xu, Weixin Zhang, Xiangfeng Meng, Guanjun Chen, Weifeng Liu

**Affiliations:** State Key Laboratory of Microbial Technology, School of Life Science, Shandong University, No.27 Shanda South Road, Jinan, 250100 Shandong People’s Republic of China

**Keywords:** MAPK, Stress response, Cell wall integrity, Cellulase production, Signal transduction, *Trichoderma reesei*

## Abstract

**Background:**

*Trichoderma reesei* holds a high capacity for protein secretion and represents the most important cellulase producer in industry. However, the external signal sensing and intracellular signal transduction during cellulose induction remain unclear. As one of the most pervasive signal transduction pathways in all eukaryotic species, the mitogen-activated protein kinase (MAPK) pathway and its upstream sensing and signaling components are involved in various physiological processes including stress and nutrient sensing. Particularly, the Hog1-type MAPK Tmk3 has been reported to be involved in the cellulase production in *T. reesei*.

**Results:**

Here we established the physiological role of two upstream regulatory branches, the Sho1 branch and the Sln1 branch, of the Hog1-type Tmk3 pathway in *T. reesei*. Deletion of *Trste20* of the Sho1 branch or repression of *Trypd1* of the Sln1 branch reduced the resistance to high salt stress, whereas TrSho1 showed an opposing effect to that of TrSte20 and the identified TrSln1 seemed to be dispensable in the osmotic regulation. The Sho1 and Sln1 branches also participated in the cell wall integrity maintenance and other stress responses (i.e. oxidative and thermo stresses). Notably, TrSho1 and TrSte20 of the Sho1 branch and TrYpd1 of the Sln1 branch were shown to be differentially involved in the cellulase production of *T. reesei*. Repression of *Trypd1* hardly affected cellulase induction, whereas overexpression of *Trypd1* resulted in the reduced production of cellulases. Contrary to the case of *Trypd1*, repression of *Trsho1* or deletion of *Trste20* significantly reduced the transcription of cellulase genes.

**Conclusions:**

TrSho1 and TrSte20 of the Sho1 branch and TrYpd1 of the Sln1 branch are all involved in general stress responses including hyperosmotic regulation and cell wall integrity maintenance. Moreover, our study revealed that the Sho1 and Sln1 osmosensing pathways are differentially involved in the regulation of cellulase production in *T. reesei*. The Sho1 branch positively regulated the production of cellulases and the transcription of cellulase genes while TrYpd1 of the Sln1 branch negatively controlled the cellulase production, supporting the crosstalks of osmosensing and nutrient sensing.

**Electronic supplementary material:**

The online version of this article (10.1186/s13068-018-1098-8) contains supplementary material, which is available to authorized users.

## Background

Lignocellulose represents the most abundant renewable resource on earth and displays a tremendous application prospect in the production of biofuel and other value-added chemicals [1]. The deconstruction of lignocellulose in an economical and environmental-friendly way is considered to be critical for their application. Cellulolytic microorganisms and their enzymes hold great potential in the degradation of recalcitrant lignocellulose [2]. Among others, the filamentous fungus *Trichoderma reesei* (telemorph *Hypocrea jecorina*) is one of the most prominent cellulase producers in nature and is widely used as a workhorse for industrial cellulase production [3]. Genetic engineering approaches, including cellulase enzyme engineering, manipulating of the enzyme composition and engineering cellulase gene expression, have been studied intensively to improve the production of cellulases [4]. Besides, new strategies for metabolic engineering in *T. reesei,* including manipulating factors that regulate carbon catabolite repression (CCR), sensing of nutrients, and chromatin remodeling, have been outlined with the aim to further increase the cellulase production in the post-genomic era [5].

Biosynthesis of cellulases by *T. reesei* involves many physiological processes, including the external signal sensing, signal transduction, transcription and translation of cellulase genes, and their secretion. The external signal sensing is the initial step of cellulase production. Both insoluble and soluble substrates including crystalline cellulose and its degradation products cellobiose as well as sophorose and lactose are capable of inducing the expression of cellulase genes in *T. reesei* [6]. However, it remains largely unknown how these substrates initiate the intracellular transduction cascade leading to the high expression of cellulase genes. Interpretation and reconstruction of these pathways could provide a new perspective and contribute to the engineering of cellulase hyper-producing strains. Among all the studied signal transduction pathways, the mitogen-activated protein kinase (MAPK) pathway is widely present in eukaryotes and plays important roles in various physiological processes [7]. Importantly, the Hog1-type MAP kinase Tmk3 was recently found to be involved in the transcriptional regulation of cellulase genes [8, 9], which implicates the participation of its upstream regulatory components in the cellulose signal transduction, hence the cellulase gene transcription process. A typical MAPK pathway is composed of a MAPK kinase kinase (MAPKKK), a MAPK kinase (MAPKK) and the MAPK [10]. When activated by sequential phosphorylation relay, the MAP kinase activates specific transcription factors which are then responsible for activating the expression of relevant target genes [11]. The most characterized MAPK pathway is the high-osmolarity glycerol (HOG) signaling pathway in *Saccharomyces cerevisiae* [12], which was identified for its role in responding to hyperosmotic shock and has been reported to be regulated by two independent upstream signaling pathways, termed the Sho1 branch and the Sln1 branch [13, 14]. In the Sho1 branch, membrane-embedded mucin proteoglycans Msb2 and Hkr1 act as osmosensor to sense osmotic stress and then interact with the membrane-anchored protein Sho1 individually, resulting in the activation of MAPKK kinase Ste11. The downstream MAPKK Pbs2 and the MAPK Hog1 are phosphorylated sequentially to activate the osmoregulation pathway [15]. Notably, Ste20 acts as a MAPKKK kinase (MAPKKKK) and is proven to be essential in the Sho1-dependent activation of Hog1 [16]. The Sln1 branch is composed of a two-component-related hybrid histidine kinase (Sln1), an intermediary phosphorelay protein (Ypd1) and a response regulator protein (Ssk1) [14]. Under low osmotic conditions, Sln1 auto-phosphorylates at His 576 and then transfers the phosphoryl group to Ssk1 through an intermediary protein Ypd1. Phosphorylated Ssk1 represses the redundant Ssk2 and Ssk22 MAPKKKs and thus the downstream Pbs2 MAPKK and Hog1 MAPK. Under high osmotic conditions, the histidine kinase activity of Sln1 is repressed, leading to the accumulation of non-phosphorylated Ssk1, which activates the Hog1 cascade [17].

Hog1 homologs are widely found in filamentous fungi which function primarily in high osmolarity resistance similarly with *S. cerevisiae*. Involvement of Hog1 homologs in other processes such as sporulation, stress response, and secondary metabolism in filamentous fungi has also been reported [9]. Interestingly, the *T. reesei* ortholog of Hog1, Tmk3, is postulated to be the only MAPK involved in signal transduction for the transcriptional regulation of cellulase genes [8]. Similar to Hog1-like MAPK, the upstream components of the cascade have been demonstrated to be extensively involved in diverse physiological processes including mediating hyperosmolarity responses. In *Neurospora crassa*, NcSte20 and two Ste20-related proteins were identified and reported to be involved in the MAK-2 pathway [18]. Moreover, a two-component system was found in *N. crassa* to play important roles in the regulation of cell wall integrity and osmotic sensing [19, 20]. In *Aspergillus nidulans*, although HogA, homolog of *S*. *cerevisiae* Hog1, was found essential for survival at high osmolarity [21], the orthologous Sho1 does not seem to be involved in osmoresponsive activation of the HogA pathway, which has been shown to be activated only by the two-component signaling pathway in response to osmotic stress [22]. Unlike *A. nidulans*, *Aspergillus fumigatus* Sho1 seems to play more important roles in growth, morphology, osmotic stress and oxidative stress sensing compared with Sln1 [23–26]. Besides the hyperosmotic response, orthologous Sho1 and Msb2 were shown to be essential virulence factors and played a key role during appressorium differentiation in the dimorphic fungus *Ustilago maydis* [27]. In *Fusarium oxysporum*, FoSho1 and FoMsb2 were found to promote invasive growth and plant infection as well as cell wall integrity [28]. More recently, the histidine kinase OS-1-mediated hyperosmotic-response pathway has even been reported to be involved in the tunable regulation of glycosyl hydrolase production in *N. crassa* in response to changes in osmolarity [29]. Deconvolution of the upstream pathway and pinpointing the differential involvement of the specific upper component in the MAPK cascade would thus contribute to understanding how saprophytic filamentous fungi including *T. reesei* accurately sense and respond to the changing environment including the nutrient state.

In this study, we aim to characterize the upstream pathways of Tmk3 and their potential involvement in regulating cellulase production. We identified two upstream regulatory branches of HOG pathway, the Sho1 branch and the Sln1 branch in *T. reesei.* By deleting TrSte20 and conditionally controlling the expression of TrSho1, TrSln1, and the intermediary phosphorelay protein TrYpd1, we characterized their roles in growth, resistance to high osmolarity, cell wall integrity maintenance and cellulase production. Our results demonstrate that these two branches play different roles in *T. reesei* in response to osmolarity and cellulase induction.

## Results

### Identification and sequence analysis of the upstream genes of HOG pathway in *T. reesei*

HOG pathway has been reported to be regulated by two independent upstream branches, the Sho1 branch and the Sln1 branch, in *S. cerevisiae* [13, 14]. To investigate whether these two branches exist in *T. reesei*, blastP was performed using HOG signaling-associated proteins of *S. cerevisiae* as queries to search the *T. reesei* genomic sequence database (https://genome.jgi.doe.gov/Trire2/Trire2.home.html). For the Sho1 branch, orthologs of yeast Sho1 (YER118C) and Ste20 (YHL007C) were identified and named TrSho1 (Tr_5466) and TrSte20 (Tr_104364), respectively. Orthologous yeast Msb2 and Hkr1 were, however, not found in *T. reesei* genome. For the Sln1 branch, orthologs of yeast Sln1 (YIL147C) and Ypd1 (YDL235C) were identified and named TrSln1 (Tr_70943) and TrYpd1 (Tr_123344), respectively.

The *Trsho1* encodes a predicted 298-amino acid protein, which showed a relatively lower identity (33%) to the Sho1 protein from *S. cerevisiae*, but a higher identity to its filamentous fungal orthologs such as that of *N. crassa* (58%), *A. nidulans* (52%), *M. oryzae* (68%) and *F. oxysporum* (70%). SMART analysis revealed that TrSho1 contains four predicted transmembrane domains near the N-terminus (amino acids 21–43, 58–77, 84–106 and 116–135, respectively), a linker domain and an SH3 domain at the C-terminus (amino acids 242–298) (Additional file 1: Fig. S2). A phylogram of the predicted Sho1 proteins further supports the close phylogenetic relationship among Sho1 orthologs in filamentous fungi (Additional file 1: Fig. S1A). Phylogenetic analysis also revealed that TrSte20 has a close evolutionary relationship with its filamentous fungal orthologs (Additional file 1: Fig. S1B), which displayed high-amino acid identity to that of *N. crassa* (60%), *A. nidulans* (81%), *M. oryzae* (59%) and *F. oxysporum* (66%), respectively. However, unlike the case of TrSho1, TrSte20 also displayed a high identity (70%) to the yeast Ste20. TrSte20 is an 821-amino acid protein containing a C-terminal catalytic domain and a PBD domain which is believed to interact with Cdc42 and Rho-like small GTPases [30] (Additional file 1: Fig. S2).

Similar to TrSho1, whereas a relatively lower identity exists between TrSln1 and ScSln1 (37%), TrSln1 shares considerable amino acid identity to the phylogenetically-related Sln1 proteins from filamentous fungi (Additional file 1: Fig. S1C), namely *N. crassa* (61%), *A. nidulans* (48%), *M. oryzae* (59%) and *F. oxysporum* (62%). TrSln1 encodes a relative large protein which contains 1170 amino acids and three predicted transmembrane domains near the N-terminus (amino acids 9–31, 233–255 and 405–427, respectively), a HisKA domain (amino acids 563–628), a HATPase histidine-like ATPase domain (705–903), and a REC signal receiver domain (999–1116) as revealed by SMART analysis (Additional file 1: Fig. S2). As the immediate phosphorelay protein downstream of Sln1, TrYpd1 encodes a relatively small protein (149 amino acids) with varied identities to the corresponding Ypd1 in *S. cerevisiae*, *N. crassa*, *A. nidulans*, *C. albicans*, *M. oryzae* and *F. oxysporum* of 39, 50, 55, 49, 58 and 69%, respectively. TrYpd1p is a putative phosphotransferase with a histidine-containing phosphotransfer domain (Additional file 1: Fig. S2). Phylogenetic analysis revealed that TrYpd1 has a close evolutionary relationship with other filamentous fungal orthologs as well (Additional file 1: Fig. S1D). Based on the above bioinformatics analyses, we believe that the upstream two branches of the Hog1-type Tmk3 exist in *T. reesei*.

### Construction of mutant strains for the Sho1 and Sln1 branches in *T. reesei* and characterization of their vegetative growth and mycelia morphology

**Fig. 1:**
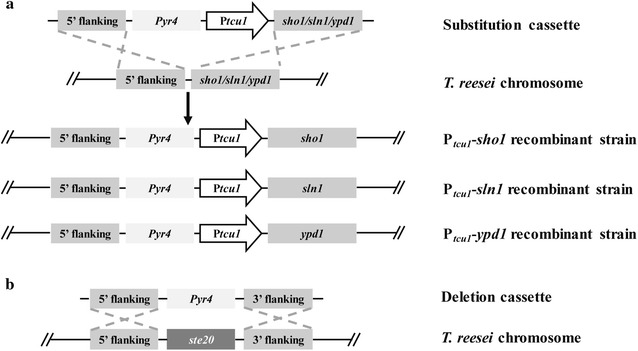
Targeted gene knockout of *Trste20* and promoter substitution of *Trsho1*, *Trsln1* or *Trypd1*. **a** Schematic illustration of the homologous integration of the *tcu1* promoter in front of the *Trsho1*, *Trsln1* or *Trypd1* gene locus. A promoter substitution cassette harboring the *tcu1* promoter and a *pyr4* marker was integrated into the *Trsho1*, *Trsln1* or *Trypd1* locus by homologous recombination. **b** Schematic representation of *Trste20* deletion. *Trste20* was replaced by the *pyr4* gene via homologous recombination

**Fig. 2:**
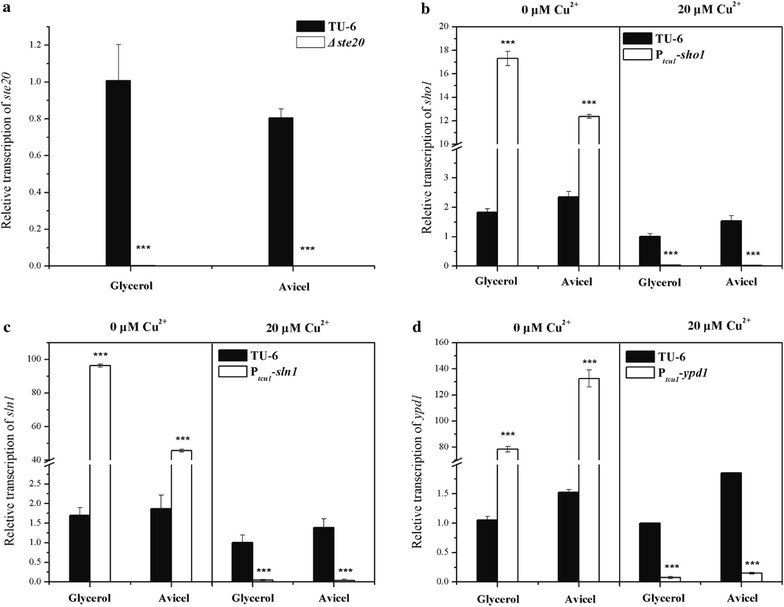
Transcriptional analyses of target genes in mutant strains. qRT-PCR analysis of the relative expression of *Trste20* (**a**), *Trsho1* (**b**), *Trsln1* (**c**) and *Trypd1* (**d**) in the parental and the mutant strains were performed under non-induced (1% glycerol) and induced (1% Avicel) conditions with and without copper. Error bars are SD from three biological replicates. *P < 0.05, **P < 0.01, ***P < 0.001

It has been reported that deletions of *sln1* or *ypd1* in *S. cerevisiae* are lethal [17]. To investigate the physiological roles of the Sho1 and Sln1 branches in *T. reesei*, particularly in hyperosmolarity response, cell wall integrity maintenance, and cellulase production that have not been studied in other fungal species, we constructed a series of promoter substitution strains of *Trsho1*, *Trsln1* and *Trypd1* using our recently developed copper-responsive promoter replacement system [31], resulting in P_*tcu1*_-*sho1*, P_*tcu1*_-*sln1* and P_*tcu1*_-*ypd1* strains, respectively (Fig. 1a). A *Trste20* deletion mutant (*Δste20*) was also constructed by homologous recombination (Fig. 1b), allowing parallel comparison of the relevant properties of the different mutants. For the promoter substitution strains, target genes controlled by P_*tcu1*_ were generally overexpressed when there’s no copper in the media. However, when copper was supplied in the media, the transcription of target genes were drastically repressed or even turned off [32], thus mimicking a knock-down/out status. The transcription level of the target genes in each promoter substitution strain was determined by qRT-PCR (Fig. 2). When copper was not added into the media, the transcription level of the target genes (*Trsho1*, *Trsln1* and *Trypd1*) in the promoter substitution strains were significantly up-regulated compared with the parental strain (5.3- to 9.5-fold for *Trsho1*, 24.5- to 57.7-fold for *Trsln1* and 74.5- to 86.9-fold for *Trypd1*). However, when 20 μM copper was added, the expression of the target genes was dramatically repressed (36.4- to 61.8-fold for *Trsho1*, 21.5- to 35.7-fold for *Trsln1* and 12.5- to 13.1-fold for *Trypd1* lower compared with the parental strain). As expected, the transcription of *Trste20* could be hardly detected in the *Δste20* strain (Fig. 2a). These data confirmed that the promoter substitution strains and the *Trste20* deletion strain were successfully constructed.

**Fig. 3:**
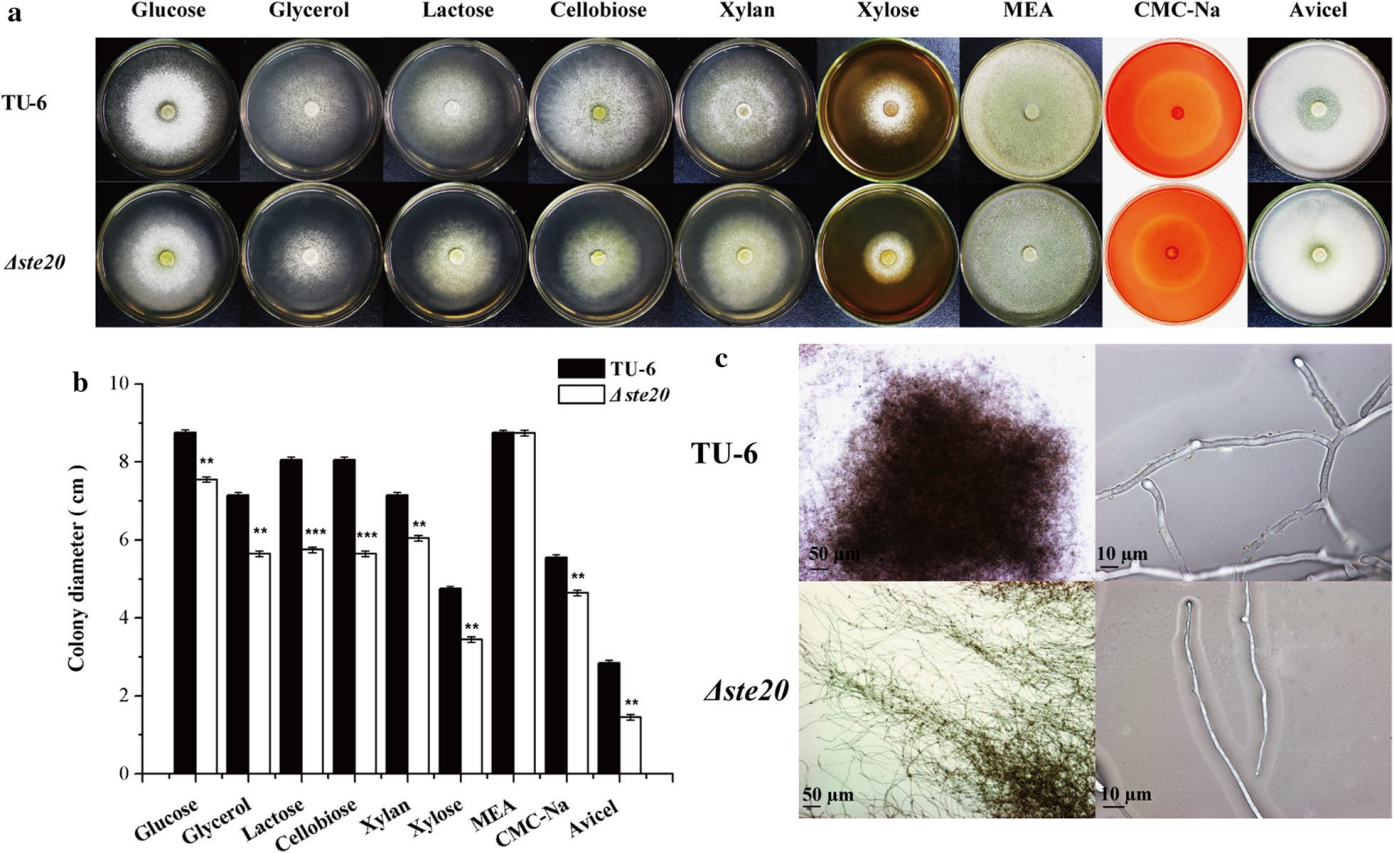
Characterization of the growth and hyphal morphology of *Δste20*. **a** Growth of TU-6 and *Δste20* strains on minimal medium plates containing different carbon sources. Strains were grown at 30 °C for 3 days. Double-layered Avicel plates were used to evaluate the crystalline cellulose hydrolysis ability. Three individual replicates of each experiment were performed. **b** Colony diameters of TU-6 and *Δste20* strains on MM plates containing different carbon source. Three individual replicates of each experiment were performed. *P < 0.05, **P < 0.01, ***P < 0.001. **c** Microscopic examination of the mycelia of TU-6 and *Δste20*. Strains were cultured for 48 h in submerged MM medium containing glucose. Scale bars were shown in the figure

**Fig. 4:**
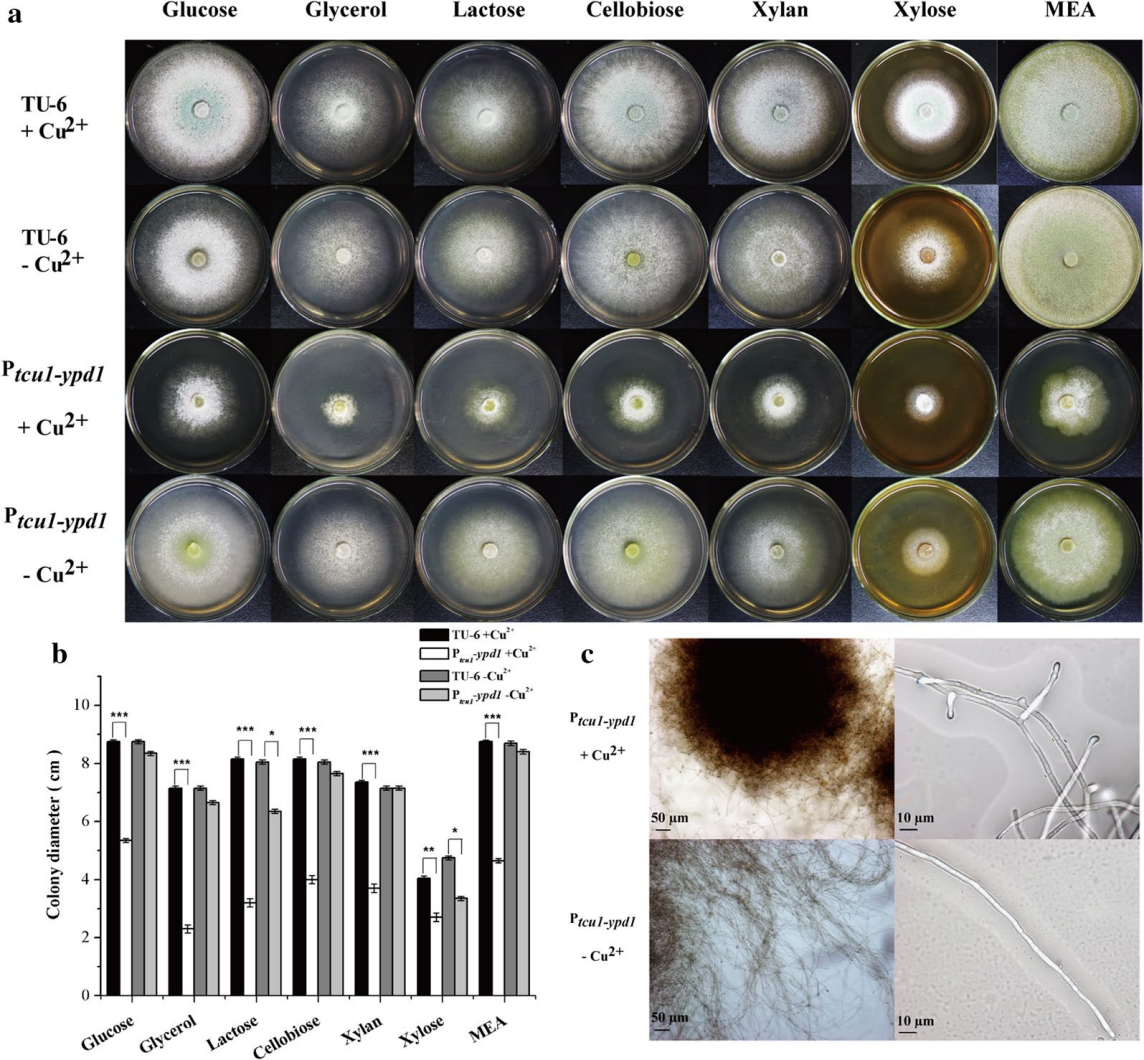
Phenotypic characterization of the growth and hyphal morphology of P_*tcu1*_-*ypd1* strain. **a** Growth of TU-6 and P_*tcu1*_-*ypd1* strains on minimal medium plates containing different carbon sources under copper-added and non-copper-added conditions. Strains were grown at 30 °C for 3 days. Three individual replicates of each experiment were performed. **b** Colony diameters of TU-6 and the P_*tcu1*_-*ypd1* strain on MM plates containing different carbon source with or without copper. Three individual replicates of each experiment were performed. *P < 0.05, **P < 0.01, ***P < 0.001. **c** Microscopic examination of P_*tcu1*_-*ypd1* mycelia cultured with and without copper. Strains were grown for 48 h in submerged MM medium containing glucose. Scale bars were shown in the figure

We first analyzed the vegetative growth of mutant strains in the Sho1 branch (*Δste20* and P_*tcu1*_-*sho1*) and in the Sln1 branch (P_*tcu1*_-*sln1* and P_*tcu1*_-*ypd1*) on different carbon sources. For the Sho1 branch, deletion of *ste20* displayed a slightly reduced growth on all the tested carbon sources on minimal medium but not on the malt extract agar plates. Notably, the *Δste20* strain displayed a significantly reduced hydrolytic zone on Avicel-containing plates compared to the parental TU-6 strain (Fig. 3a, b), suggesting the involvement of *ste20* in regulating cellulase production in *T. reesei*. We further examined the hyphal morphology of *Δste20* and TU-6 strains. The mycelia of TU-6 were relatively highly branched and formed relatively compact clots in submerged culture, whereas the *Δste20* strain displayed vimineous mycelia with significantly reduced branches resulting in much loose mycelia aggregates (Fig. 3c). In contrast to the absence of TrSte20, no obvious change could be detected in the growth, sporulation and hyphal morphology of P_*tcu1*_-*sho1* (Additional file 1: Fig. S3 and data not shown), regardless the presence or absence of copper which has been proven to result in the repression or overexpression of the target gene placed under the control of P_*tcu1*_ [31]. The copper ions had no significant effect on the growth and sporulation of the parent strain TU-6 (Fig. 4a).

For the Sln1 branch, the *Trsln1* promoter-substituted strain P_*tcu1*_-*sln1* showed no obvious difference in growth and sporulation no matter whether copper was added or not (Additional file 1: Fig. S3). In contrast, the growth and sporulation of the P_*tcu1*_-*ypd1* strain were severely impaired under copper-repressing conditions, whereas no significant difference was observed compared to the parental strain when copper was excluded in the media (Fig. 4a, b). Microscopic examination revealed that highly branched hyphal morphology was observed with the repression of *Trypd1,* whereas overexpression of *Trypd1* without copper resulted in a less-branched hyphal morphology (Fig. 4c).

### Characterization of the functional roles of *Trste20*, *Trsho1*, *Trsln1* and *Trypd1* in different stress responses of *T. reesei*

**Fig. 5:**
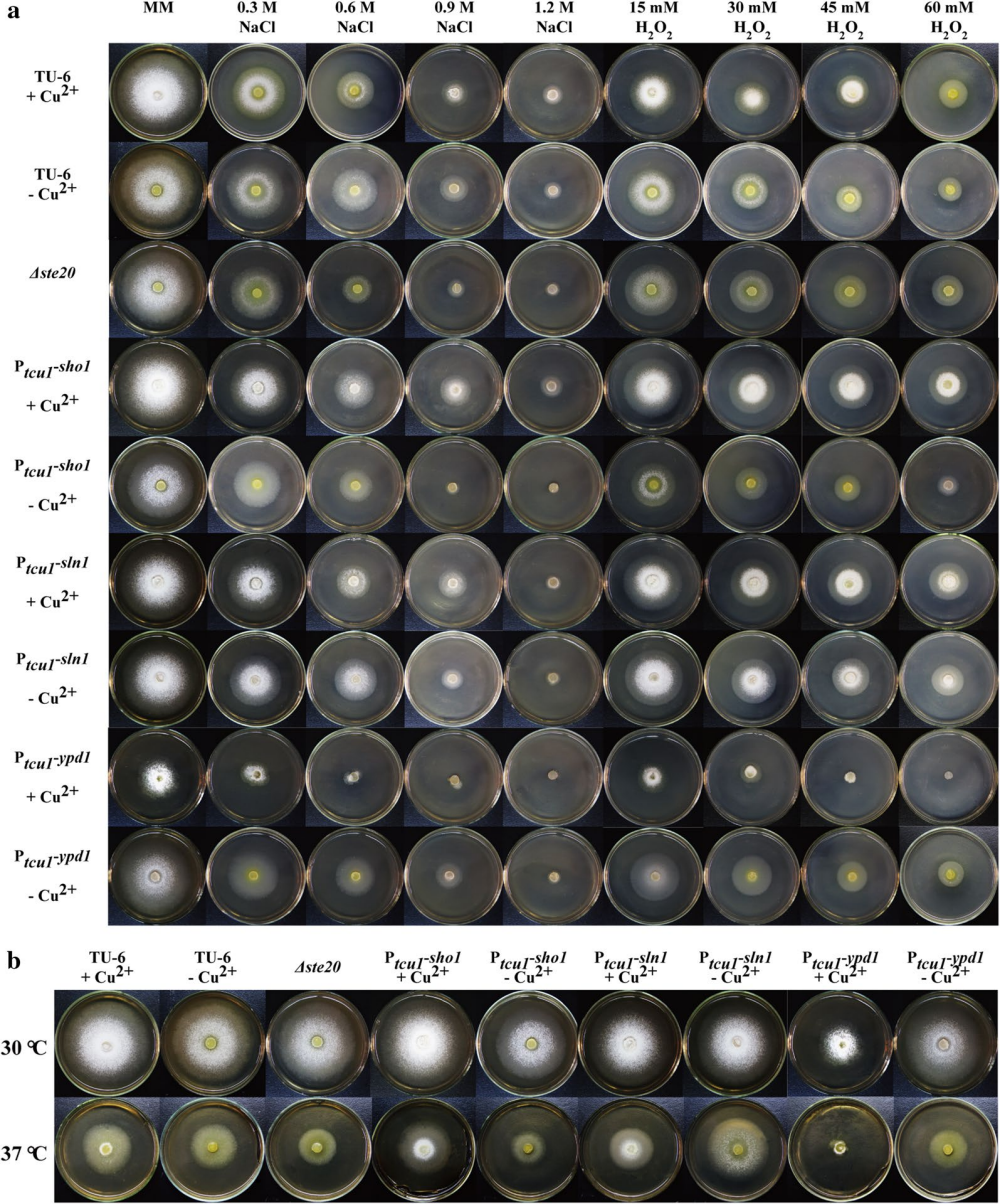
Different stress response tests of the parental TU-6 and mutant strains. **a** Osmotic stress and oxidative stress tests were performed by adding different concentrations of NaCl and H_2_O_2_ to the MM plates, respectively. All the strains were grown at 30 °C for 3 days. **b** Growth of TU-6 and mutant strains on MM plates at 30 and 37 °C for 3 days, respectively

Since the Hog1-related kinases have been shown to primarily participate in osmotic stress response [12], we examined the tolerance of the mutant and parental strains to different stresses. To investigate their roles in high osmolarity resistance, all the strains were grown on MM plates containing various concentrations of NaCl from 0.3 M to 1.2 M. Meanwhile, oxidative stress response was analyzed by including different concentrations of H_2_O_2_ from 15 to 60 mM. In addition, thermotolerance was also tested by growing all the strains at 37 °C. As expected, deletion of *Trste20* in the Sho1 branch caused a clear growth defect under high salt stress (0.6 M NaCl and above) or at high temperature although hardly any effect was observed on the resistance to oxidative stress (Fig. 5 and Additional file 1: Fig. S4A). In contrast, when repressed with copper, the P_*tcu1*_-*sho1* strain showed slightly enhanced growth under high salt (0.6 M NaCl and above) and oxidative stress (15 mM H_2_O_2_ and above) compared to the parental strain (Fig. 5 and Additional file 1: Fig. S4B). In corroborating the above results, the P_*tcu1*_-*sho1* strain displayed decreased tolerance to high salt, H_2_O_2_ and higher temperature (37 °C) when overexpressed without copper.

The involvement of the Sln1 branch in stress responses was also investigated by growing the P_*tcu1*_-*sln1* and P_*tcu1*_-*ypd1* strains on MM plates under the respective stress conditions. TrSln1 exerted hardly any effect on growth under all the tested stress conditions compared to the parental strain no matter whether it was repressed or de-repressed (Fig. 5 and Additional file 1: Fig. S4C). However, the P_*tcu1*_-*ypd1* strain appeared extremely sensitive to all the tested stresses when the expression of *Trypd1* was repressed with copper. Specifically, the P_*tcu1*_-*ypd1* strain could hardly grow on plates containing 0.6 M NaCl or 45 mM H_2_O_2_ under copper-repressed conditions (Fig. 5 and Additional file 1: Fig. S4D). When copper was not included, the P_*tcu1*_-*ypd1* strain showed no significant growth difference on different stress plates compared to the parental TU-6 strain. The apparently highest sensitivity exhibited by the *Trypd1*-repressed strain is an indication of hampered tolerance to the above stress conditions, suggesting that aberrantly activated HOG-like MAP kinase cascade may compromise *T. reesei* tolerance to stresses.

### The effect of *Trste20*, *Trsho1*, *Trsln1* and *Trypd1* on cell wall integrity maintenance of *T. reesei*

**Fig. 6:**
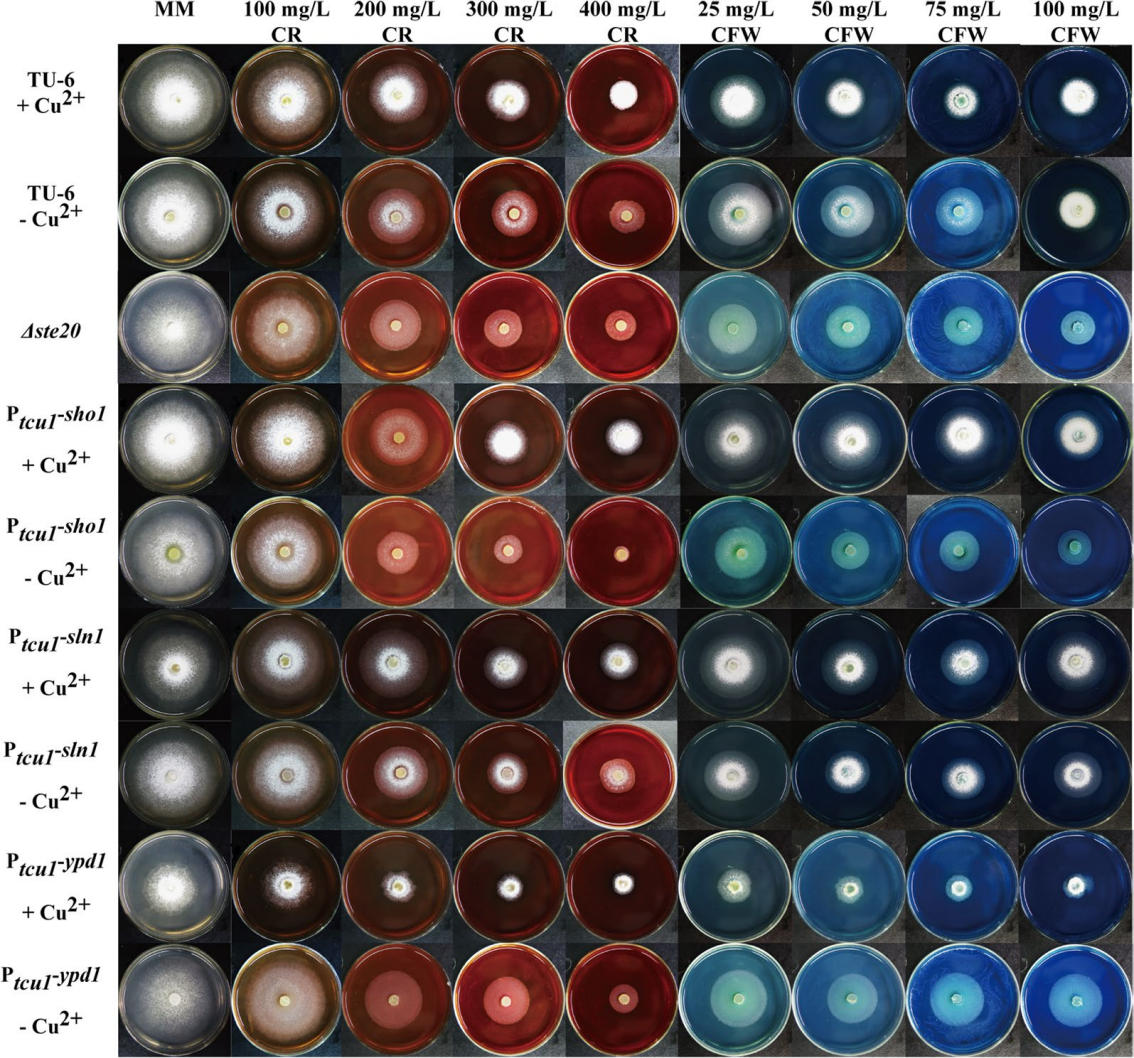
The effect of *Trste20*, *Trsho1*, *Trsln1* and *Trypd1* on cell wall integrity maintenance. Different concentrations of Calcofluor white (CFW) or Congo red (CR) were added to the minimal medium (MM) plates containing 1% glucose as the sole carbon source. Strains were grown at 30 °C for 3 days

In addition to the involvement in different stress responses, Tmk3 was also found to participate in the cell wall integrity maintenance [8]. Besides, given the fact that the Sho1 and Sln1 branches differentially affect the response to various stresses, we analyzed their participation in cell wall integrity maintenance by testing the sensitivity of the mutant and parental strains to Calcofluor white (CFW) and Congo red (CR), which has been proven to be indicators of cell wall integrity [33]. For the Sho1 branch, contrary to our expectation, the *Δste20* strain displayed no obvious difference in the sensitivity to both CFW and CR compared to the parental strain (Fig. 6 and Additional file 1: Fig. S5A). Similar to the case in tolerance to stresses, repression of *Trsho1* with copper slightly increased in its resistance to CFW and CR while growth of the P_*tcu1*_-*sho1* strain in the absence of copper was compromised compared to that of TU-6 when the concentration of CR or CFW raised to higher than 200 or 50 mg/l. Specifically, the P_*tcu1*_-*sho1* strain could hardly grow at 400 mg/ml CR without copper (Fig. 6 and Additional file 1: Fig. S5B). Together these data suggested that, unlike *TrSte20*, *Trsho1* plays a negative role in cell wall integrity maintenance, which may provide an explanation for its opposing role to that of TrSte20 in mediating stress responses.

In the case of the P_*tcu1*_-*sln1* strain, no significant difference in the sensitivity to both CFW and CR was observed with and without copper between the P_*tcu1*_-*sln1* and TU-6 parental strains (Fig. 6 and Additional file 1: Fig. S5C). In contrast, P_*tcu1*_-*ypd1* showed severely compromised growth on plates with CR or CFW higher than 100 or 25 mg/l in the presence of copper, respectively (Fig. 6 and Additional file 1: Fig. S5D), indicating that repression of *Trypd1* showed impaired cell wall integrity. When de-repressed without copper, P_*tcu1*_-*ypd1* strain displayed a slightly increased tolerance to both CFW and CR. These results implicated that *Trypd1* plays a critical role in the cell wall integrity maintenance in *T. reesei*.

### Differential involvement of *Trste20*, *Trsho1*, *Trsln1* and *Trypd1* in *T. reesei* cellulase production

**Fig. 7:**
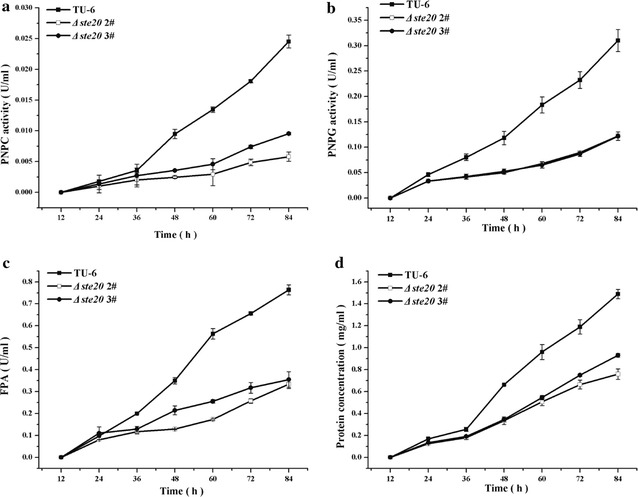
Deletion of *Trste20* reduced cellulase production. Extracellular hydrolytic activities of *p*NPC (**a**), *p*NPG (**b**), FPA (**c**) and protein concentration (**d**) of the parental and the *Δste20* strains were analyzed as described in the material and method section. All the strains were cultured in Mandels–Andreotti medium containing 1% (w/v) Avicel as the sole carbon source

**Fig. 8:**
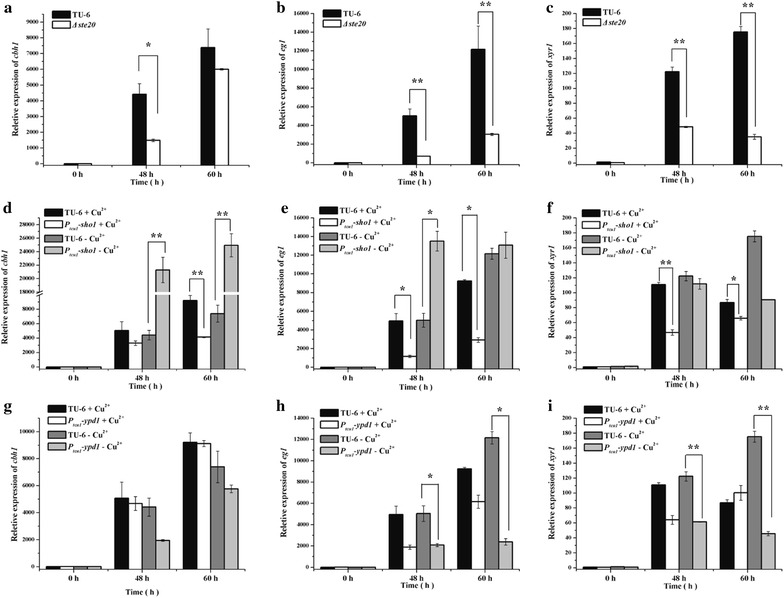
Transcriptional analysis of *chb1*, *eg1* and *xyr1* in different mutant and parental strains. Relative transcription levels of the endogenous *cbh1*, *eg1* and *xyr1* of the *Δste20* strain (**a**–**c**), the P_*tcu1*_-*sho1* strain (**d**–**f**) and the P_*tcu1*_-*ypd1* strain (**g**–**i**) were determined. All strains were cultured on 1% (w/v) Avicel supplied with or without 20 μM CuSO_4_. *P < 0.05, **P < 0.01

Previous studies revealed that although deletion of *tmk2* increased the cellulase production, minor difference could be detected in the transcription level of cellulase genes, indicating that *tmk2* was not directly involved in cellulase production [34]. The Hog1-type MAPK Tmk3 is the only MAPK involved in signal transduction for regulating cellulase expression on the transcriptional level in *T. reesei* [8]. Specifically, in submerged cultivation condition, deletion of *tmk3* significantly down-regulated the transcription of the main cellulase genes, including *cbh1*, *chb2*, *eg1*, *eg2* and *bgl1* [8]. Characterizing the functional roles of the Tmk3 upstream pathways in cellulase production is thus important in the understanding of external signal sensing and signal transduction in *T. reesei*. Different cascade mutants and the parental strain were cultivated in Mandels–Andreotti medium supplied with 1% Avicel as the sole carbon source. The extracellular hydrolytic activities (*p*NPC and *p*NPG) and the filter paper activity (FPA) were analyzed. Furthermore, the mRNA levels of two major cellulase genes (*cbh1* and *eg1*) and the major transcription activator *xyr1* were analyzed by quantitative RT-PCR. As shown in Fig. 7a–c, the *p*NPC and *p*NPG hydrolytic activities and the filter paper activity (FPA) of the *Δste20* strain were dramatically reduced compared with the parental strain, in accordance with the heavily reduced hydrolytic zone on the Avicel plates (Fig. 3a). Meanwhile, the total extracellular protein concentration was also significantly decreased (Fig. 7d). Moreover, the transcriptional level of *chb1*, *eg1* and *xyr1* in *Δste20* was significantly lower than that of the TU-6 strain (Fig. 8a–c), suggesting that the reduced cellulase production resulted from the impaired cellulase gene transcription. These results indicated that *Trste20* is critical for the efficient induction of cellulase gene expression in *T. reesei*.

**Fig. 9:**
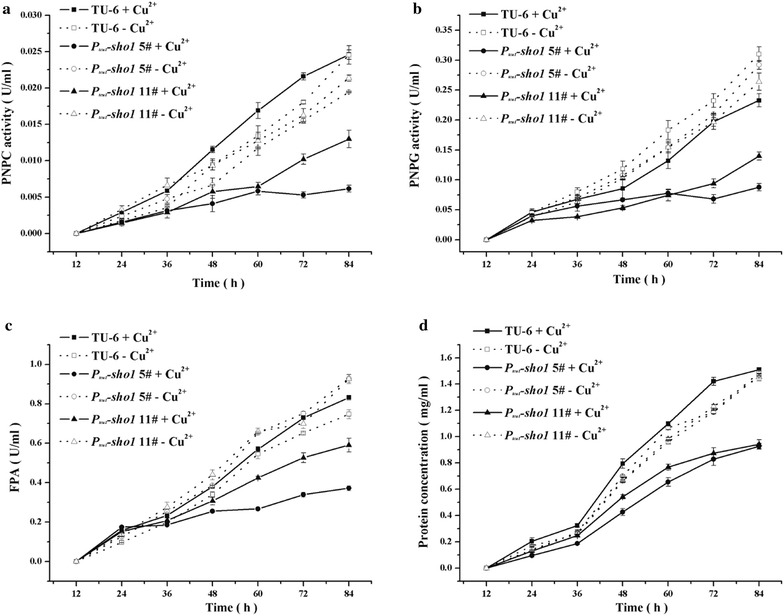
Copper-repressed *Trsho1* caused severe defect on cellulase production. Extracellular hydrolytic activities of *p*NPC (**a**), *p*NPG (**b**), FPA (**c**), and total protein concentration (**d**) of the parent strain and the P_*tcu1*_-*sho1* strain were determined. All strains were cultured on 1% (w/v) Avicel supplied with or without CuSO_4_

In accordance with *Δste20*, when copper was added to a final concentration of 20 μM to repress the expression of *Trsho1*, the extracellular *p*NPC and *p*NPG hydrolytic activities decreased to 25–50% those of the parental TU-6 strain (Fig. 9a, b). FPA and the total extracellular protein concentration also showed a clear decline (Fig. 9c, d). Further transcriptional analysis revealed that repression of *Trsho1* significantly reduced the transcription level of *cbh1*, *eg1* and *xyr1* (Fig. 8d–f). No significant differences were observed with these hydrolytic activities and the extracellular protein concentration when *Trsho1* was not repressed with copper. The transcriptional level of *chb1* and *eg1* was even up-regulated compared to the parental strain in the absence of copper. Together, these data indicate that similar to *Trste20*, *Trsho1* is critical for the transcription of cellulase genes, suggesting that the Sho1 branch is conductive to cellulase production.

**Fig. 10:**
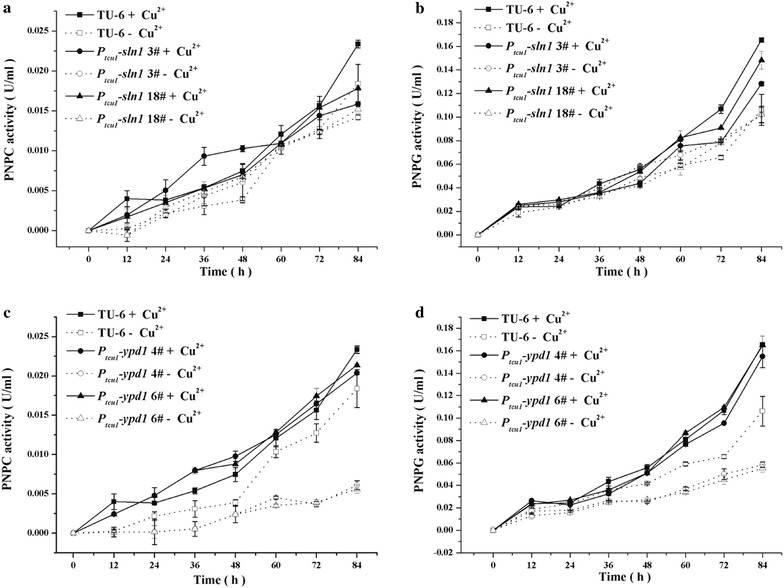
Influence of *Trsln1* and *Trypd1* on cellulase production. Extracellular hydrolytic activities of *p*NPC (**a**, **c**) and *p*NPG (**b**, **d**) of the P_*tcu1*_-*ypd1* strain, the P_*tcu1*_-*sln1* strain and the parent strain were determined. All strains were cultured on 1% (w/v) Avicel supplied with or without CuSO_4_

Unlike the Sho1 branch, the two Sln1 branch components exhibited different effects on cellulase production by *T. reesei*. For the P_*tcu1*_-*sln1* strain, the extracellular *p*PNC and *p*NPG hydrolytic activities maintained at a similar level to those of the parental strain regardless of the presence or absence of copper (Fig. 10a, b). Correspondingly, no significant changes were detected in the transcription of *chb1* and e*g1* under either copper-included or copper-free conditions (data not shown), indicating that *Trsln1* plays a minor role in mediating cellulase gene expression. In accordance with TrSln1, repression of *Trypd1* with copper exerted hardly any effect on the extracellular *p*PNC and *p*NPG hydrolytic activities (Fig. 10c, d). On the contrary, de-repression of *Trypd1* in the absence of copper resulted in a significant reduction of the extracellular *p*NPC and *p*NPG hydrolytic activities of the P_*tcu1*_-*ypd1* strain to about 25% those of the TU-6 strain. Further transcriptional analysis demonstrated that a significant decrease was observed in the transcription level of *cbh1*, *eg1* and *xyr1* in the P_*tcu1*_-*ypd1* strain when copper was excluded in the medium (Fig. 8g–i). Together, these data suggested that, in contrast to its role in mediating stress tolerance, the hyperosmotic-response MAP kinase pathway fine-tuned by TrYpd1 may play a subtle role in the cellulolytic response in *T. reesei*.

## Discussion

Over 20 years ago, Hog1 was first identified in yeast to mediate osmosensing and signal transduction [35]. As the core component of the stress-activated protein kinase (SAPK) pathways, Hog1 has been shown to play a central role in stress-activated signaling [12]. Further studies revealed that two upstream branches are responsible for the activation of Hog1-mediated SAPK pathway, termed the Sho1 branch and the Sln1 branch [13, 14]. The HOG cascade together with its upstream components are pervasive in other filamentous fungi although each specific component plays differential roles in various filamentous fungi [26, 36–39], implicating a possible evolutionary divergence.

As one of the most prolific cellulase producers, *T. reesei* is widely used in industry and well-studied, especially in the transcriptional regulation of cellulase genes. Many transcription factors are characterized such as the transcription activator Xyr1 and the catabolite repressor Cre1 [40, 41]. However, studies focusing on the upstream initiating events leading to cellulase gene expression, such as extracellular signal sensing and intracellular signal transduction, are relatively rare. Three MAPKs are identified recently in *T. reesei* and the Hog1-type Tmk3 has been shown to be involved in cellulase expression at the transcriptional level [8]. This reminds us that tracing up to its upstream activating pathways may contribute to our understanding how the putative nutrient-sensing network of *T. reesei* transmits information about available carbon sources to tightly regulate the expression of cellulase genes. In our present research, genes involved in the Sho1 branch and the Sln1 branch were identified in *T. reesei* with considerable identities to their homologous genes in yeast and other filamentous fungi, suggesting that the upstream pathways of the Hog1-related MAPK are evolutionarily conserved in these filamentous fungi and may play important roles. However, homologs of yeast Msb2 and Hkr1, two putative osmosensors in the Sho1 branch, were not identified in *T. reesei*. In *U. maydis* and *F. oxysporum*, orthologs of Msb2 were present and shown to be involved not only in mediating a general stress response to a variety of stresses, but also in the pathogenesis process [27, 28]. Therefore, how *T. reesei* senses the osmotic stress in the absence of Msb2- and Hkr1-like osmosensors remains unknown. Unlike its orthologs in other fungi [36], deletion of *ste20* in *T. reesei* caused only a slight growth defect on different carbon sources and did not affect the sporulation. But, as expected, deletion of *Trste20* caused a decrease in resistance to high salt stress and thermotolerance although no obvious effect on oxidative stress and cell wall integrity was observed. Ste20 has been shown to be essential in the Sho1-dependent activation of Hog1 in *S. cerevisiae* [16]. Surprisingly, TrSho1 seemed to play a negative role in mediating stress responses since repression of *Trsho1* seemed to increase their resistance to various stresses and cell wall integrity maintenance, whereas overexpression of *Trsho1* displayed an opposite effect. Although the precise mechanism warrants further study, our present results indicate the opposing roles of TrSho1 and TrSte20 in mediating the various stress responses of *T. reesei*.

Histidine kinases are known to transmit information of environmental stimuli through signal-transduction pathways. The two-component hybrid histidine kinase Sln1 was identified to be partially responsible for regulating the activation of Hog1 in *S. cerevisiae* [14]. Deletion of *ypd1* or *sln1* resulted in the constitutive activation of Hog1 and consequently led to lethality [17]. However, *ypd1* homologs are not essential in *C. albicans* and *M. oryzae*, suggesting its divergent roles in different fungi [42, 43]. Here in our research, orthologs of the two most upstream components of the Sln1 branch were also identified in *T. reesei*. Repression of *Trypd1* in *T. reesei* caused a severe growth defect, indicating that TrYpd1 may play similar functional roles to those in *S. cerevisiae.* Similar to the deletion of *ste20*, overexpression of *ypd1* in *T. reesei* also affected the morphology of mycelia with fewer branches. Notably, our results revealed that repression of *Trypd1* severely compromised the resistance to high salt stress, oxidative stress, and high temperature. Moreover, repression of *Trypd1* reduced the resistance to CFW and CR while its overexpression displayed an enhanced resistance, suggesting that *Trypd1* plays a positive role in different stress responses and in the maintenance of cell wall integrity possibly by acting as a brake of hyperosmotic responses upon prolonged stressing and is essential for gating of osmotic responses. In contrast to *Trypd1*, the repression or overexpression of the identified *Trsln1* had no obvious effect on the growth, hyphal morphology, and resistance to various stresses tested. Similar phenotypic characteristics of the *sln1* deletion strains have been reported in other fungi. In *C. albicans*, deletion of *sln1* is viable and does not significantly affect the ability to adapt to osmotic stress [44]. In *A. nidulans*, *tcsB* deletion strain was viable under both regular and high osmotic conditions [45]. One possible explanation for the dispensable roles of TrSln1 in mediating general responses including hyperosmotic response may lie in the fact that more than one Sln1 paralog exist in *T. reesei* and many other filamentous fungi, whereas Sln1 is the sole sensor histidine kinase in the *S. cerevisiae* proteome [26]. There are at least three putative histidine kinases in *C. albicans* and *S. pombe* [44, 46–48]. In *C. neoformans*, *M. oryzae* and *A. nidulans*, the number of putative histidine kinase is 7, 10 and 15, respectively [49–51]. In *T. reesei*, except TrSln1, we also found nine other putative histidine kinases. SMART analysis revealed that all these Sln1-like proteins contain a HisKA domain, a HATPase histidine-like ATPase domain and a REC signal receiver domain. However, only the TrSln1 has three transmembrane domains and other Sln1-like proteins may locate in the cytoplasm. Since there is only one intermediary phosphorelay protein (Ypd1) in *T. reesei*, these ten putative Sln1-like proteins may interact with TrYpd1 independently and thus transduce different signals.

Previous studies demonstrated that all three identified MAPKs in *T. reesei*, namely Tmk1, Tmk2 and Tmk3, affect the cellulase production but through different mechanisms [9]. Only Hog1-type Tmk3 is directly involved in regulating the transcription of cellulase genes in *T. reesei*. In the present study, we investigated the functions of the upstream components of the Tmk3 cascade in cellulase production to elucidate the carbon source signal transduction processes in *T. reesei*. Our results revealed that the Sho1 and Sln1 branches are differentially involved in regulating cellulase production. Deletion of *Trste20* or repression of *Trsho1* caused severe reduction in both cellulase formation and cellulase gene transcription, indicating that the Sho1 branch is critical for the full induction of cellulase gene expression. It is worth noting that, although TrSho1 and TrSte20 exerted opposing effects on the mycelia morphology and stress response, they seem to play similar roles in the cellulase production in *T. reesei*. One assumption is that an uncoupled intermediate signal transduction pathway, which acts downstream of TrSho1 but parallel to TrSte20, independently modulates cellulase production. In contrast to the Sho1 branch, the absence of *Trypd1* displayed hardly any effect on cellulase induction although it resulted in the severest defect in stress responses. Considering that *ypd1* negatively controlled Hog1 since deletion of *ypd1* or *sln1* caused constitutively activation of Hog1 [17], one might surmise that an unconstrained activated MAPK cascade is not necessary for the full induction of cellulase synthesis. However, taking into account that either overexpression of *Trypd1* or deletion of *Trste20* reduced the cellulase production, it is reasonable to believe that appropriate activation of the Tmk3 cascade plays an important role in transmitting the cellulase-inducing signals. It was recently reported that, working together with other pathways, the hyperosmotic-response pathway is involved in forming a nutrient-sensing network that allows the tunable regulation of glycosyl hydrolase production in response to changes in osmolarity in *N. crassa* [29]. Although it is suggested that osmotic defect is partially responsible for the increased expression of plant cell wall-degrading enzymes under non-inducing conditions with de-repressed (hemi)cellulolytic responses [29], our results did not show such a correlation between osmotic defect and an enhanced cellulolytic response. Nonetheless, our results indeed support the note that there is a crosstalk between osmosensing and nutrient sensing in filamentous fungi. Either perturbation or enforcement of these nutrient-sensing network signaling pathways thus holds great potential to increase the production of those plant cell wall-degrading enzymes by unlocking the tight regulation exerted on gene expression in response to environmental conditions. On the other hand, orchestrating the high-tolerance of the strain to various stresses with its high producing capabilities is critical for production of cellulases in industry. Our present study may contribute to constructing a cellulase hyper-producing strain with high-tolerance to harsh conditions (e.g. high salt and high temperature). It will be interesting for future studies to identify the specific transcription factor(s) or downstream effectors that are activated by the osmotic response pathway and to elucidate the mechanistic differences in cellulolytic filamentous fungi regarding how the osmotic response pathway is implicated in their cellulolytic responses.

## Conclusions

**Fig. 11:**
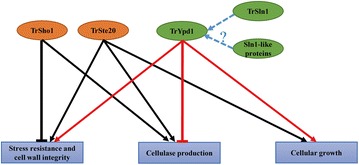
Overview of different physiological processes regulated by the Sho1 and Sln1 branches. TrSte20 of the Sho1 branch positively contributes to the cellular growth, osmosensing and cellulase production. However, TrSho1 of the Sho1 branch negatively controls the osmosensing but benefits cellulase production. TrYpd1 of the Sln1 branch is favorable for growth, different stress sensing and cell wall integrity maintenance but detrimental for the cellulase production. Since the identified TrSln1 is dispensable, other Sln1-like proteins may take part in the activation of TrYpd1

In conclusion, we established the physiological roles of the Sho1 branch (TrSho1 and TrSte20) and the Sln1 branch (TrSln1 and TrYpd1) in the prolific cellulase producer *T. reesei.* A full picture of the roles played by the Sho1 branch and the Sln1 branch on cellular growth, cellulase production, different stress sensing and cell wall integrity in *T. reesei* can be generated based on our study (Fig. 11). For the Sho1 branch, deletion of *Trste20* caused an obvious defect in the osmotic response, whereas repression of *Trsho1* increased the resistance to osmotic, oxidative and thermo stresses. In addition, TrSho1 was also shown to be involved in the cell wall integrity maintenance. For the Sln1 branch, whereas repression or overexpression of the identified *Trsln1* caused minor effect on growth and stress responses, repression of *Trypd1* significantly impaired the vegetative growth, cell wall integrity maintenance and stress responses.

Besides, TrSho1 and TrSte20 of the Sho1 branch and TrYpd1 of the Sln1 branch were also shown to be differentially involved in the cellulase production of *T. reesei*. Specifically, repression of *Trsho1* or deletion of *Trste20* significantly reduced the transcription of cellulase genes whereas overexpression of *Trypd1* resulted in the reduced production of cellulases. These results indicate that the Sho1 branch and the Sln1 branch oppositely regulate the cellulase production in *T. reesei*. Our present study implicates the osmotic response pathways in regulating the cellulase production, supporting the crosstalks of osmosensing and nutrient sensing. Our research thus provides insight into the cellulose signal transduction process and contributes to our understanding of the physiological network of regulation in response to environmental cues in *T. reesei*.

## Methods

### Strains, media and cultivation conditions

*Escherichia coli* DH5α was used for plasmid construction and amplification. The uridine-auxotrophic strain *T. reesei* TU-6 (ATCC MYA-256) was used as a parental strain for recombinant strain construction throughout this work. All *T. reesei* strains were maintained on malt extract agar (Sigma-Aldrich, Madison, USA) supplemented with 10 mM uridine when necessary. For cellulase transcription and production analysis, *T. reesei* strains were pre-cultured in 1-l Erlenmeyer flasks on a rotary shaker (200 rpm) at 30 °C in 250 ml Mandels–Andreotti medium with glycerol (1%, v/v) as the carbon source for 36 h and further grown for another 12 h in the same fresh medium. Mycelia were harvested by filtration and washed twice with fresh Mandels–Andreotti medium with no carbon source. Equal amounts of mycelia were then transferred to a fresh medium containing 1% (w/v) Avicel (Sigma-Aldrich, Madison, USA), and incubation was continued for the indicated times.

### Phylogenetic analysis and protein domain annotation

Homologous proteins of Sho1, Ste20, Sln1 and Ypd1 in *T. reesei* were obtained from NCBI (http://www.ncbi.nlm.nih.gov/) by blastP search using their homologous protein sequences in *S. cerevisiae* as query sequences. Sequence alignments were performed by MUSCLE and phylogenetic tree were constructed with MEGA6 using the Maximum Likelihood method with 1000 bootstraps. Protein domain annotation was obtained using SMART (http://smart.embl-heidelberg.de/). Prediction of transmembrane helices was performed using TMHMM (http://www.cbs.dtu.dk/services/TMHMM-2.0/).

### Plasmids and recombinant *T. reesei* strain construction

To delete *ste20*, two approximately 2.0-kb DNA fragments of *ste20* upstream and downstream non-coding regions were amplified from TU-6 genomic DNA and ligated into pDONOR-*pyr*4 via BP-cloning (Invitrogen) to yield the disruption vector pDONOR-*ste20*-*pyr*4, which was used to transform *T. reesei* after linearization with I-SceI. The pDONOR-*pyr*4 plasmid was obtained as described previously [52]. To construct the *sho1* promoter replacement vector, the 2.0-kb upstream flanking sequence and 2.0-kb fragment starting from the initiation code ATG of the *sho*1 were amplified from the genomic DNA of TU-6, digested with *Hin*dIII/*Asc*I and *Not*I/*Spe*I, respectively, and ligated into the corresponding sites of pMD-P_*tcu1*_-*pry*4 sequentially to obtain pMD-P_*tcu1*_-*sho*1. Similarly, the 2.2-kb upstream region of the *sln1*/*ypd1* encoding sequence digested by *Hin*dIII/*Asc*I and the 2.0-kb fragment containing the *sln1*/*ypd1* ORF region digested by *Not*I/*Spe*I were ligated into pMD-P_*tcu1*_-*pyr*4 to obtain pMD-P_*tcu1*_-*sln1* and pMD-P_*tcu1*_-*ypd1*, respectively. The pMD-P_*tcu1*_-*pyr*4 plasmid was obtained as described previously [31].

Fungal transformation was performed as described by Penttila et al. [53]. Transformants were selected on the minimal medium for uridine prototroph. Anchored PCR was performed to verify the correct integration events.

### Vegetative growth assay

To compare the vegetative growth of the recombinant strains and the parent strain, equal amounts of growing mycelia of each strain were inoculated on minimal medium (MM) agar plates containing different carbon source (1% glucose, 1% glycerol, 1% cellobiose, 1% lactose, 1% xylan, 1% xylose, 0.5% CMC-Na and 0.5% Avicel) or on mart extract agar plates at 30 °C for 3 days. When it is necessary, 20 μM CuSO_4_ was added in the media. For the examination of hydrolyzation zone in CMC-Na-containing plates, 1 mg/ml Congo red was added into the plates and incubated for 15 min, then washed three times with 1 M NaCl for 15 min.

### Sensitivity test to CR, CFW, NaCl and H_2_O_2_

To test the cell wall integrity and the sensitivity under different stress conditions, the mutant and the parent strains were inoculated on MM plates with 1% (w/v) glucose as the sole carbon source with or without 20 μM copper. Various stress conditions were applied by including 15, 30, 45, 60 mM H_2_O_2_ for oxidative stress, 0.3, 0.6, 0.9, 1.2 M NaCl for osmotic pressure, 37 °C for high temperature stress, 100, 200, 300, 400 mg/l Congo red and 25, 50, 75, 100 mg/ml CFW for the test of cell wall integrity. After incubation for 3 days, the mycelia diameter under the indicated conditions was recorded. Three replicates of each experiment were performed.

### Microscopic analyses

To visualize the hyphal morphology difference between the mutant and parental strains, equal amounts of spores were inoculated and cultured in MM liquid medium containing 1% (w/v) glucose for 36 h at 30 °C with shaking at 200 rpm. After incubation, fungal hyphae were spread onto glass slides and imaged with a Nikon Eclipse 80i microscope (Nikon, Melville, NY, USA). Images were captured and processed using the NIS-ELEMENTSAR software.

### Enzyme activity and protein analysis

The activities of cellobiohydrolases (*p*NPCase) and β-glucosidases (*p*NPGase) were determined by measuring the amount of released *p*-nitrophenol using *p*-nitrophenyl-β-d-cellobioside (*p*NPC; Sigma) and *p*-nitrophenyl-β-d-glucopyranoside (*p*NPG; Sigma) as substrates, respectively. The filter paper activity (FPA) was determined by measuring the released reducing sugar with Whatman No. 1 filter paper as the substrate. The *p*NPC and *p*NPG activity assays were performed in 200 μl reaction mixtures containing 50 μl of culture supernatant and 50 μl of the respective substrate plus 100 μl of 50 mM sodium acetate buffer (pH 4.8) and then incubated at 45 °C for 30 min. One unit (U) of *p*NPCase or *p*NPGase activity was defined as the conversion of 1 μmol of substrate per minute under the test conditions. The FPA assay was performed at 50 °C in 200 μl reaction mixture including 50 μl of appropriately diluted culture supernatant and 150 μl 50 mM sodium acetate buffer (pH 4.8). One unit (U) of FPA was defined as the release of 1 μmol reducing sugar per minute under the test conditions. Total secreted proteins were determined using the Bradford protein assay with bovine serum albumin (BSA) as a standard. At least two biological replicates were carried out for each experiment.

### Nucleic acid isolation and quantitative RT-PCR (qRT-PCR)

Fungal genomic DNA was extracted according to the instructions of E.Z.N.A.™ fungal DNA miniprep kit (Omega Biotech, Doraville, USA). Trizol reagent (Invitrogen, USA) was used for total RNA isolation according to the manufacturer’s protocol and then a Turbo DNA-free kit (Ambion) was used to digest and eliminate genomic DNA contamination. For reverse transcription (RT), a PrimeScript RT reagent kit (TaKaRa, Japan) was applied according to the manufacturer’s instructions. Quantitative real-time PCRs were performed using SYBR green supermix (TaKaRa, Japan) on a qTOWER^3^ thermocycler (Analytik Jena, Germany). Reactions were performed in triplicates with a total volume of 20 μl, including 250 nM (each) forward and reverse primers and template cDNA. Data were analyzed using the relative quantitation/comparative threshold cycle (ΔΔCT) method and were normalized to an endogenous gene *actin*1. Three biological replicates were performed for each experiment. Two-tailed Student’s *t*-tests were used to test the significant difference between two sets of data. *P* < 0.05 was considered to be significant different. A minimum of three replicates were present in each set of data.

## Additional file

**Additional file 1.** Additional figures.

## Abbreviations

P_*tcu1*_: *tcu1* promoter
*p*NPC: *p*-nitrophenyl-β-d-cellobioside
*p*NPG: *p*-nitrophenyl-β-d-glucopyranoside
FPA: filter paper activities
CMC: carboxymethylcellulose sodium salt

## References

[1] Kubicek CP, Kubicek EM (2016). Enzymatic deconstruction of plant biomass by fungal enzymes. Curr Opin Chem Biol.

[2] Bomble YJ, Lin CY, Amore A, Wei H, Holwerda EK, Ciesielski PN, Donohoe BS, Decker SR, Lynd LR, Himmel ME (2017). Lignocellulose deconstruction in the biosphere. Curr Opin Chem Biol.

[3] Bischof RH, Ramoni J, Seiboth B (2016). Cellulases and beyond: the first 70 years of the enzyme producer *Trichoderma reesei*. Microb Cell Fact.

[4] Druzhinina IS, Kubicek CP (2017). Genetic engineering of *Trichoderma reesei* cellulases and their production. Microb Biotechnol.

[5] Gupta VK, Steindorff AS, de Paula RG, Silva-Rocha R, Mach-Aigner AR, Mach RL, Silva RN (2016). The post-genomic Era of *Trichoderma reesei*: what’s next?. Trends Biotechnol.

[6] Suto M, Tomita F (2001). Induction and catabolite repression mechanisms of cellulase in fungi. J Biosci Bioeng.

[7] Banuett F (1998). Signalling in the yeasts: an informational cascade with links to the filamentous fungi. Microbiol Mol Biol Rev.

[8] Wang M, Zhao Q, Yang J, Jiang B, Wang F, Liu K, Fang X (2013). A mitogen-activated protein kinase Tmk3 participates in high osmolarity resistance, cell wall integrity maintenance and cellulase production regulation in *Trichoderma reesei*. PLoS ONE.

[9] Wang M, Zhang M, Li L, Dong Y, Jiang Y, Liu K, Zhang R, Jiang B, Niu K, Fang X (2017). Role of *Trichoderma reesei* mitogen-activated protein kinases (MAPKs) in cellulase formation. Biotechnol Biofuels.

[10] Cargnello M, Roux PP (2011). Activation and function of the MAPKs and their substrates, the MAPK-activated protein kinases. Microbiol Mol Biol Rev.

[11] Hohmann S (2009). Control of high osmolarity signalling in the yeast *Saccharomyces cerevisiae*. FEBS Lett.

[12] Brewster JL, Gustin MC (2014). Hog 1: 20 years of discovery and impact. Sci Signal.

[13] Maeda T, Takekawa M, Saito H (1995). Activation of yeast PBS2 MAPKK by MAPKKKs or by binding of an SH3-containing osmosensor. Science.

[14] Posas F, Wurgler-Murphy SM, Maeda T, Witten EA, Thai TC, Saito H (1996). Yeast HOG1 MAP kinase cascade is regulated by a multistep phosphorelay mechanism in the SLN1-YPD1-SSK1 “two-component” osmosensor. Cell.

[15] Tatebayashi K, Tanaka K, Yang HY, Yamamoto K, Matsushita Y, Tomida T, Imai M, Saito H (2007). Transmembrane mucins Hkr1 and Msb2 are putative osmosensors in the SHO1 branch of yeast HOG pathway. EMBO J.

[16] Raitt DC, Posas F, Saito H (2000). Yeast Cdc42 GTPase and Ste20 PAK-like kinase regulate Sho1-dependent activation of the Hog1 MAPK pathway. EMBO J.

[17] Horie T, Tatebayashi K, Yamada R, Saito H (2008). Phosphorylated Ssk1 prevents unphosphorylated Ssk1 from activating the Ssk2 mitogen-activated protein kinase kinase kinase in the yeast high-osmolarity glycerol osmoregulatory pathway. Mol Cell Biol.

[18] Dettmann A, Heilig Y, Valerius O, Ludwig S, Seiler S (2014). Fungal communication requires the MAK-2 pathway elements STE-20 and RAS-2, the NRC-1 adapter STE-50 and the MAP kinase scaffold HAM-5. PLoS Genet.

[19] Alex LA, Borkovich KA, Simon MI (1996). Hyphal development in *Neurospora crassa*: involvement of a two-component histidine kinase. Proc Natl Acad Sci USA.

[20] Schumacher MM, Enderlin CS, Selitrennikoff CP (1997). The *osmotic*-*1* locus of *Neurospora crassa* encodes a putative histidine kinase similar to osmosensors of bacteria and yeast. Curr Microbiol.

[21] Han KH, Prade RA (2002). Osmotic stress-coupled maintenance of polar growth in *Aspergillus nidulans*. Mol Microbiol.

[22] Furukawa K, Hoshi Y, Maeda T, Nakajima T, Abe K (2005). *Aspergillus nidulans* HOG pathway is activated only by two-component signalling pathway in response to osmotic stress. Mol Microbiol.

[23] Ma Y, Qiao J, Liu W, Wan Z, Wang X, Calderone R, Li R (2008). The Sho1 sensor regulates growth, morphology, and oxidant adaptation in *Aspergillus fumigatus* but is not essential for development of invasive pulmonary aspergillosis. Infect Immun.

[24] Yang F, Ma D, Wan Z, Liu W, Ji Y, Li R (2011). The role of Sho1 in polarized growth of *Aspergillus fumigatus*. Mycopathologia.

[25] Du C, Sarfati J, Latge JP, Calderone R (2006). The role of the *sakA (Hog1)* and *tcsB (sln1)* genes in the oxidant adaptation of *Aspergillus fumigatus*. Med Mycol.

[26] Ma D, Li R (2013). Current understanding of HOG-MAPK pathway in *Aspergillus fumigatus*. Mycopathologia.

[27] Lanver D, Mendoza-Mendoza A, Brachmann A, Kahmann R (2010). Sho1 and Msb2-related proteins regulate appressorium development in the smut fungus *Ustilago maydis*. Plant Cell.

[28] Perez-Nadales E, Di Pietro A (2015). The transmembrane protein Sho1 cooperates with the mucin Msb2 to regulate invasive growth and plant infection in *Fusarium oxysporum*. Mol Plant Pathol.

[29] Huberman LB, Coradetti ST, Glass NL (2017). Network of nutrient-sensing pathways and a conserved kinase cascade integrate osmolarity and carbon sensing in *Neurospora crassa*. Proc Natl Acad Sci USA.

[30] Lamson RE, Winters MJ, Pryciak PM (2002). Cdc42 regulation of kinase activity and signaling by the yeast p21-activated kinase Ste20. Mol Cell Biol.

[31] Zheng F, Cao Y, Lv X, Wang L, Li C, Zhang W, Chen G, Liu W (2017). A copper-responsive promoter replacement system to investigate gene functions in *Trichoderma reesei*: a case study in characterizing SAGA genes. Appl Microbiol Biotechnol.

[32] Lv X, Zheng F, Li C, Zhang W, Chen G, Liu W (2015). Characterization of a copper responsive promoter and its mediated overexpression of the xylanase regulator 1 results in an induction-independent production of cellulases in *Trichoderma reesei*. Biotechnol Biofuels.

[33] Ram AF, Klis FM (2006). Identification of fungal cell wall mutants using susceptibility assays based on Calcofluor white and Congo red. Nat Protoc.

[34] Wang M, Dong Y, Zhao Q, Wang F, Liu K, Jiang B, Fang X (2014). Identification of the role of a MAP kinase Tmk2 in *Hypocrea jecorina* (*Trichoderma reesei*). Sci Rep.

[35] Brewster JL, de Valoir T, Dwyer ND, Winter E, Gustin MC (1993). An osmosensing signal transduction pathway in yeast. Science.

[36] Boyce KJ, Andrianopoulos A (2011). Ste20-related kinases: effectors of signaling and morphogenesis in fungi. Trends Microbiol.

[37] de Dios CH, Roman E, Monge RA, Pla J (2010). The role of MAPK signal transduction pathways in the response to oxidative stress in the fungal pathogen *Candida albicans*: implications in virulence. Curr Protein Pept Sci.

[38] Kruppa M, Calderone R (2006). Two-component signal transduction in human fungal pathogens. FEMS Yeast Res.

[39] Hirt H (2000). MAP kinases in plant signal transduction. Results Probl Cell Differ.

[40] Strauss J, Mach RL, Zeilinger S, Hartler G, Stoffler G, Wolschek M, Kubicek CP (1995). Cre1, the carbon catabolite repressor protein from *Trichoderma reesei*. FEBS Lett.

[41] Rauscher R, Wurleitner E, Wacenovsky C, Aro N, Stricker AR, Zeilinger S, Kubicek CP, Penttila M, Mach RL (2006). Transcriptional regulation of *xyn1*, encoding xylanase I, in *Hypocrea jecorina*. Eukaryot Cell.

[42] Jacob S, Foster AJ, Yemelin A, Thines E (2015). High osmolarity glycerol (HOG) signalling in *Magnaporthe oryzae*: identification of *MoYPD1* and its role in osmoregulation, fungicide action, and pathogenicity. Fungal Biol.

[43] Mavrianos J, Desai C, Chauhan N (2014). Two-component histidine phosphotransfer protein Ypd1 is not essential for viability in *Candida albicans*. Eukaryot Cell.

[44] Nagahashi S, Mio T, Ono N, Yamada-Okabe T, Arisawa M, Bussey H, Yamada-Okabe H (1998). Isolation of *CaSLN1* and *CaNIK1*, the genes for osmosensing histidine kinase homologues, from the pathogenic fungus *Candida albicans*. Microbiology.

[45] Furukawa K, Katsuno Y, Urao T, Yabe T, Yamada-Okabe T, Yamada-Okabe H, Yamagata Y, Abe K, Nakajima T (2002). Isolation and functional analysis of a gene, *tcsB*, encoding a transmembrane hybrid-type histidine kinase from *Aspergillus nidulans*. Appl Environ Microbiol.

[46] Calera JA, Choi GH, Calderone RA (1998). Identification of a putative histidine kinase two-component phosphorelay gene (*CaHK1*) in *Candida albicans*. Yeast.

[47] Yamada-Okabe T, Mio T, Ono N, Kashima Y, Matsui M, Arisawa M, Yamada-Okabe H (1999). Roles of three histidine kinase genes in hyphal development and virulence of the pathogenic fungus *Candida albicans*. J Bacteriol.

[48] Catlett NL, Yoder OC, Turgeon BG (2003). Whole-genome analysis of two-component signal transduction genes in fungal pathogens. Eukaryot Cell.

[49] Bahn YS, Kojima K, Cox GM, Heitman J (2006). A unique fungal two-component system regulates stress responses, drug sensitivity, sexual development, and virulence of *Cryptococcus neoformans*. Mol Biol Cell.

[50] Motoyama T, Ochiai N, Morita M, Iida Y, Usami R, Kudo T (2008). Involvement of putative response regulator genes of the rice blast fungus *Magnaporthe oryzae* in osmotic stress response, fungicide action, and pathogenicity. Curr Genet.

[51] Hagiwara D, Mizuno T, Abe K (2009). Characterization of NikA histidine kinase and two response regulators with special reference to osmotic adaptation and asexual development in *Aspergillus nidulans*. Biosci Biotechnol Biochem.

[52] Zhang W, Kou Y, Xu J, Cao Y, Zhao G, Shao J, Wang H, Wang Z, Bao X, Chen G, Liu W (2013). Two major facilitator superfamily sugar transporters from *Trichoderma reesei* and their roles in induction of cellulase biosynthesis. J Biol Chem.

[53] Penttila M, Nevalainen H, Ratto M, Salminen E, Knowles J (1987). A versatile transformation system for the cellulolytic filamentous fungus *Trichoderma reesei*. Gene.

