# Supplementary material for: Functional characterization of the upstream components of the Hog1-like kinase cascade in hyperosmotic and carbon sensing in Trichoderma reesei
Source: Biotechnol Biofuels. 2018 Apr 4;11:97. doi: 10.1186/s13068-018-1098-8 (PMC5883349; doi:10.1186/s13068-018-1098-8)
Supplement: Supplementary file 1 — Additional file 1. Additional figures. [file 13068_2018_1098_MOESM1_ESM.pdf]

Additional file

Functional characterization of the upstream components of the Hog1-like kinase cascade in hyperosmotic and carbon sensing in *Trichoderma reesei*

Zhixing Wang, Ning An, Wenqiang Xu, Weixin Zhang, Xiangfeng Meng*, Guanjun Chen, Weifeng Liu*

No.27 Shanda South Road, State Key Laboratory of Microbial Technology, School of Life Science, Shandong University, Jinan 250100, Shandong, P. R. China

Corresponding to Xiangfeng Meng and Weifeng Liu; email: x.meng@sdu.edu.cn; weifliu@sdu.edu.cn.

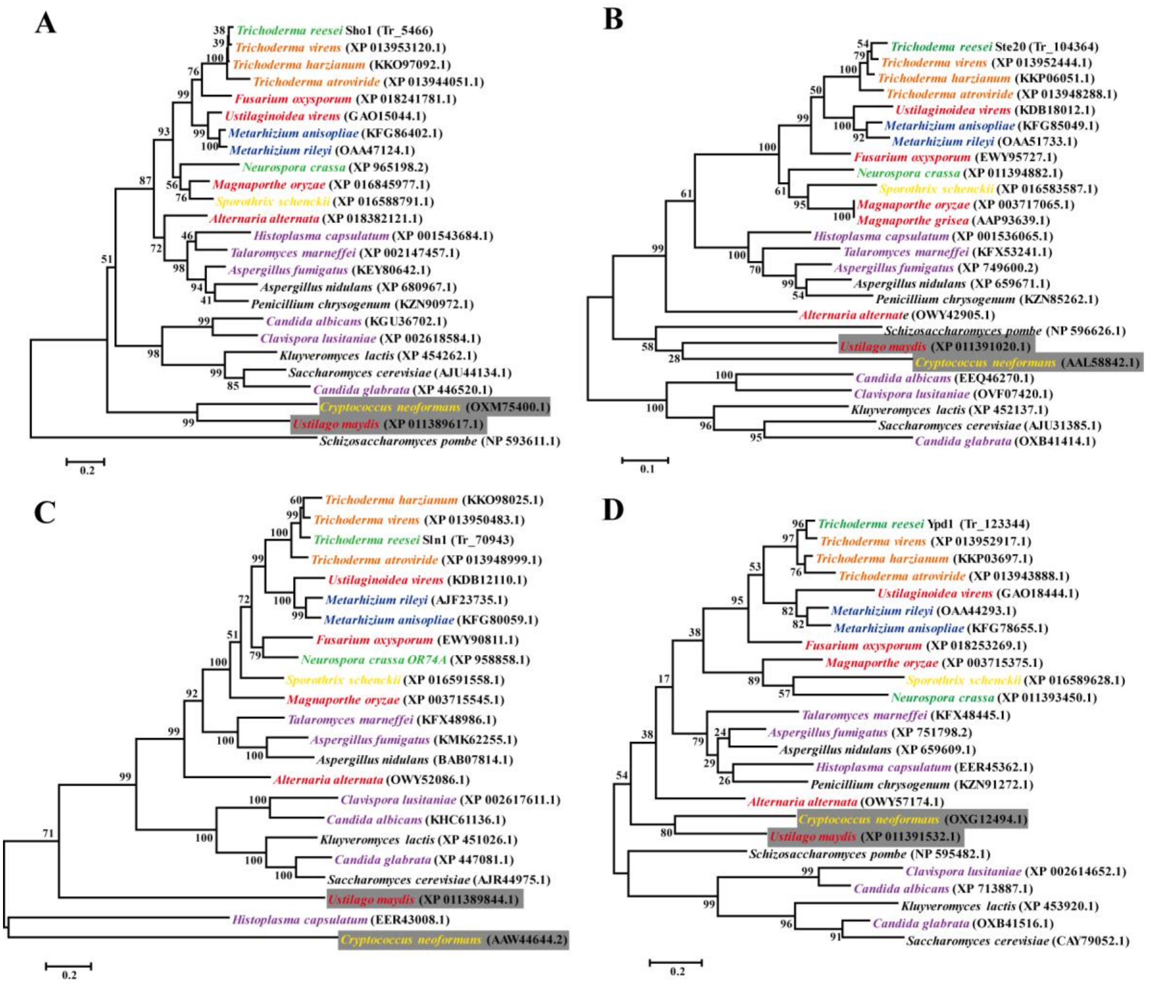

Figure S1. Phylogenetic analyses of TrSho1 (A), TrSte20 (B), TrSln1 (C) and TrYpd1 (D) with their homologs. Sequence alignments were performed by MUSCLE, and the evolutionary relationship was inferred by using the Maximum Likelihood method based on the JTT matrix-based model. Numbers on the tree branches represent the bootstrap support calculated per 1000 bootstrap replicates. Evolutionary analyses were conducted in MEGA6. Strains in green indicate these are cellulolytic fungi. Strains in orange indicate these fungi hold the ability for biocontrol. Strains in red indicate these are plant pathogens. Strains in blue indicate these are fungal pathogens whose host are insects. Strains in purple indicate these are fungal pathogens whose host are human or animals. Strains in yellow indicate these are fungal pathogens whose host can be plant or human. Strains in grey backguound are basidiomycota, the other strains are all ascomycota.

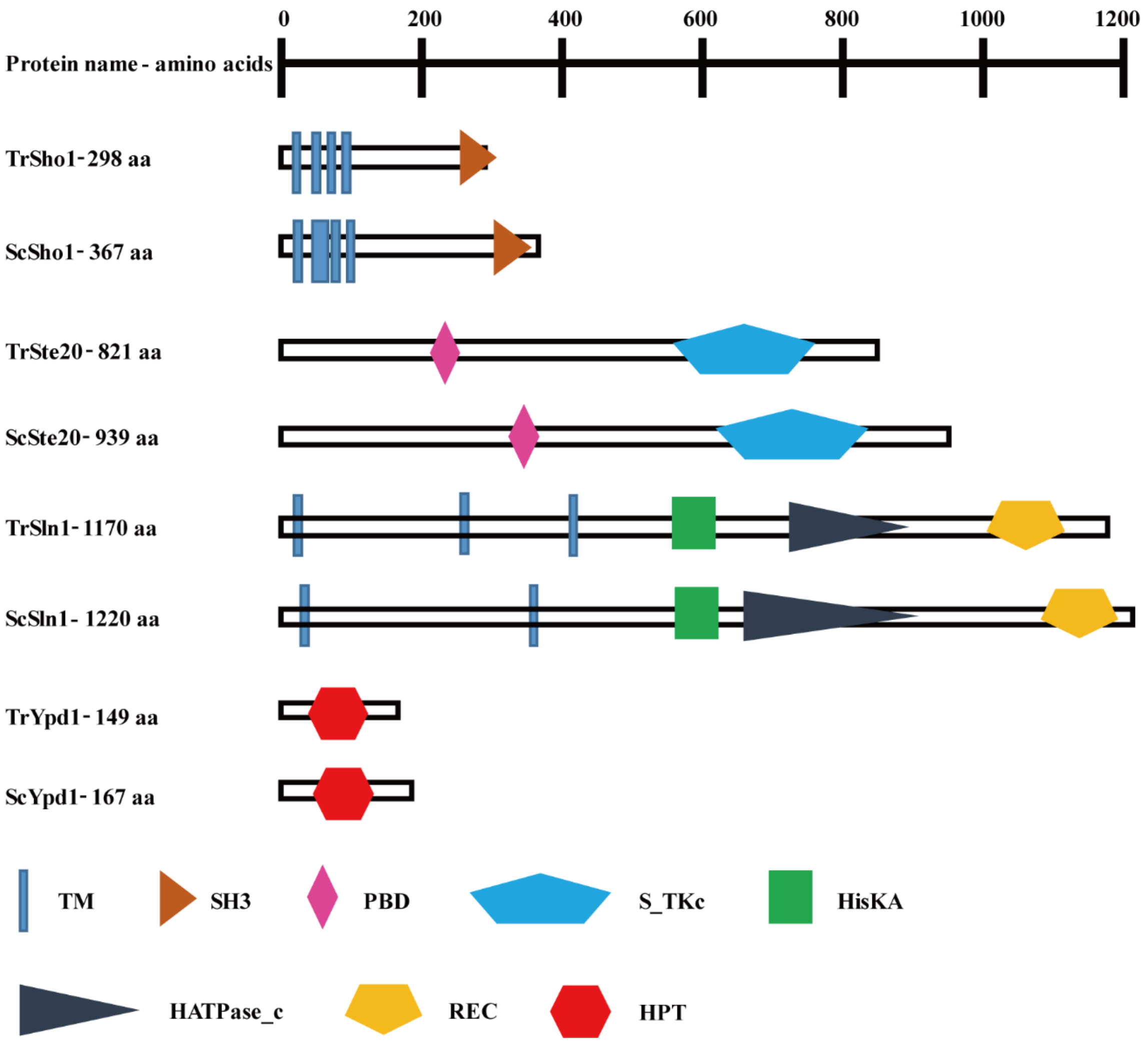

Figure S2. Schematic presentation of the protein domains of TrSte20, TrSho1, TrSln1 and TrYpd1 and their homologs in *Saccharomyces cerevisiae*. Protein domain annotation was obtained using SMART (http://smart.embl-heidelberg.de/). Prediction of transmembrane helices was performed using TMHMM (http://www.cbs.dtu.dk/services/TMHMM-2.0/). TM: transmembrane domain, SH3: Src homology 3 domains, PBD: P21-Rho-binding domain, S_TKc: Serine/Threonine protein kinases, catalytic domain, HisKA: His Kinase A (phosphoacceptor) domain, HATPase_c: Histidine kinase-like ATPases, REC: cheY-homologous receiver domain, HPT: Histidine Phosphotransfer domain.

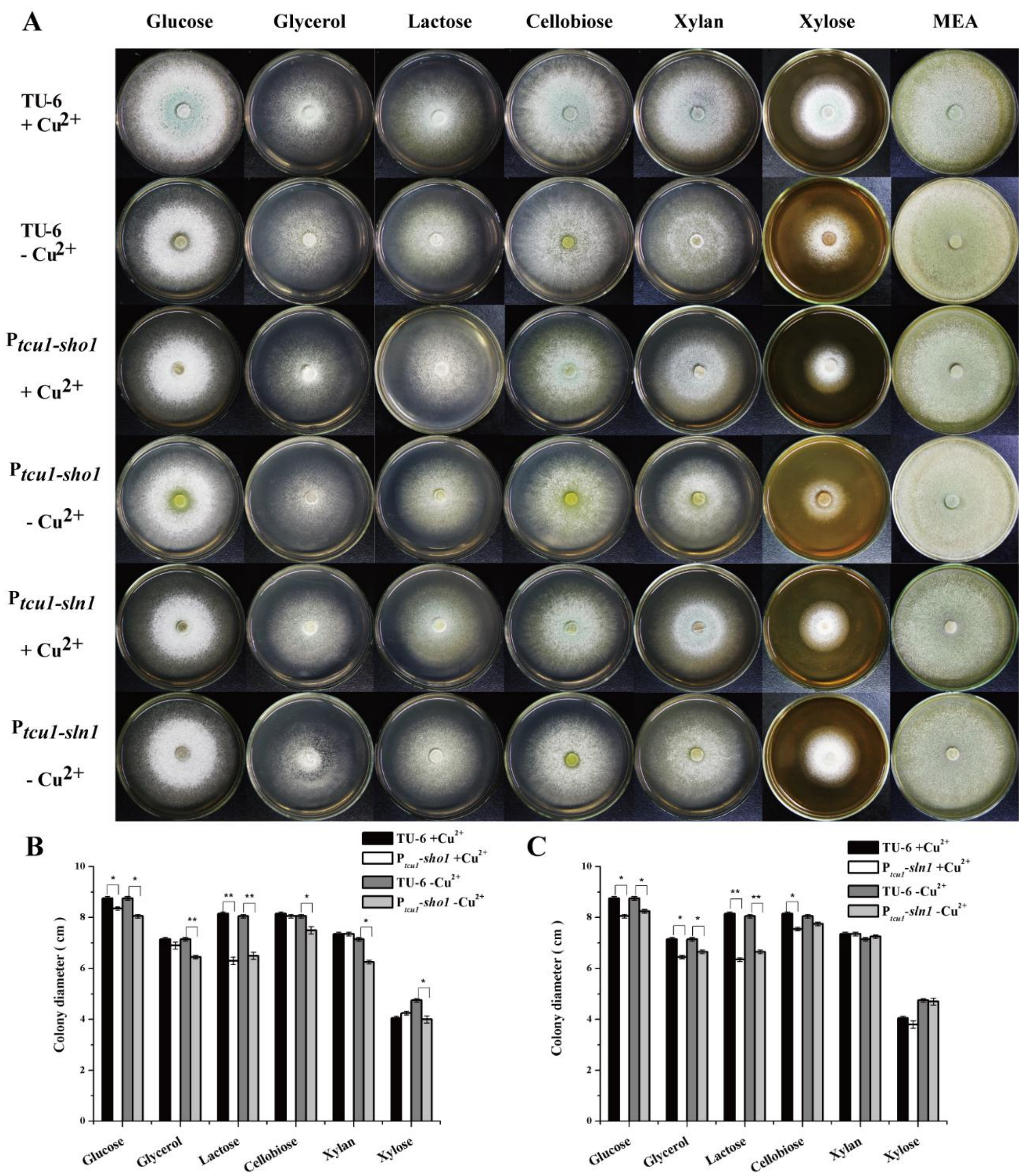

Figure. S3. **Growth and sporulation of WT, the P$_{tcu1}$-*sho1* strain and the P$_{tcu1}$-*sln1* strain.** (A) Growth of TU-6, P$_{tcu1}$-*sho1* and P$_{tcu1}$-*sln1* strains on minimal medium plates containing different carbon source in copper added and non-copper added conditions. Strains were grown at 30 ℃ for 3 days. Colony diameters of TU-6, P$_{tcu1}$-*sho1* (B) and P$_{tcu1}$-*sln1* (C) on MM plates containing different carbon source with or without copper. * $P<0.05$, ** $P<0.01$, *** $P<0.001$.

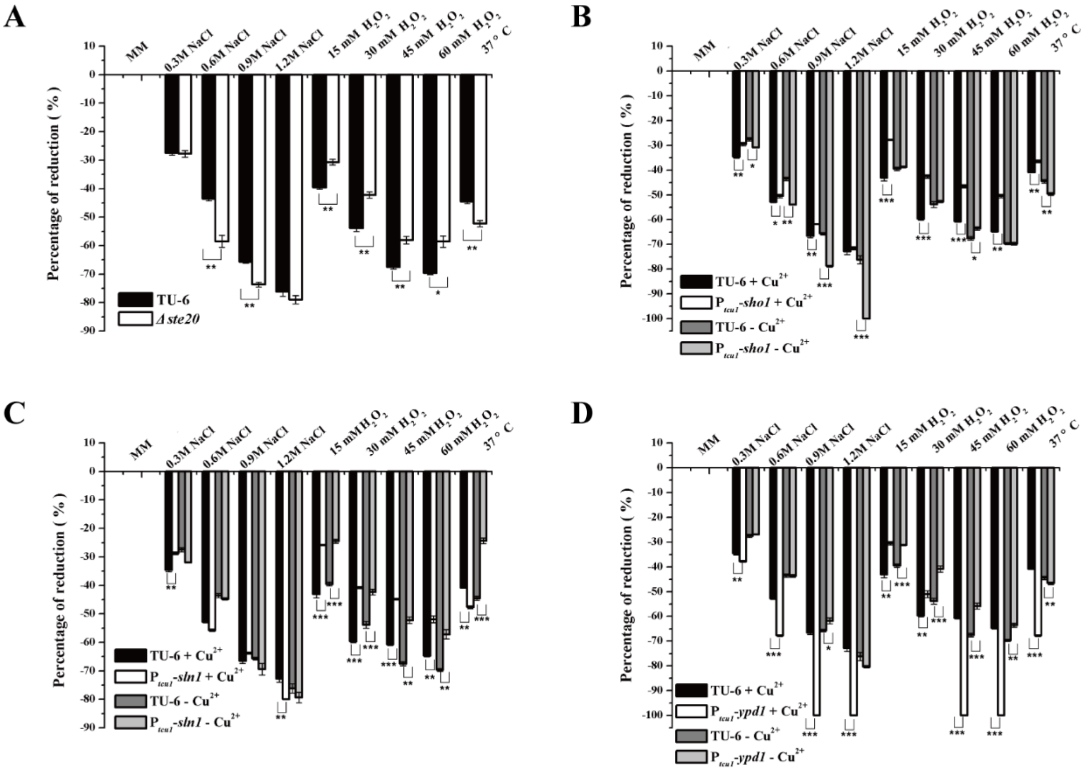

Figure. S4 **Reduction growth of WT and mutant strains under different stresses.** Statistical quantitation of the ratio of reduction in mycelia diameter for each strain under the indicated stress conditions. The WT versus the *Δste20* strain (A), the WT versus the P_{tcu1}-*sho1* strain (B), the WT versus the P_{tcu1}-*sln1* strain (C) and the WT versus the P_{tcu1}-*ypd1* strain (D). * $P<0.05$, ** $P<0.01$, *** $P<0.001$.

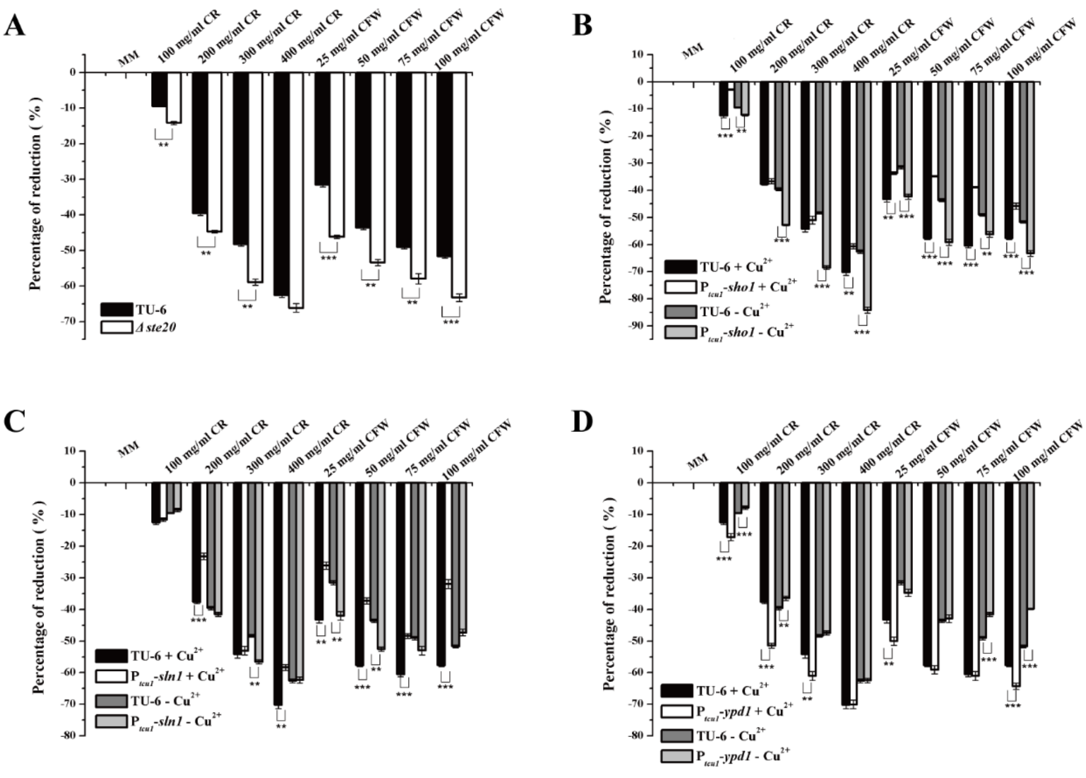

Figure. S5 **Reduction growth of WT and mutant strains on MM plates containing different concentration of CR or CFW.** Statistical quantitation of the ratio of reduction in mycelia diameter for each strain under different concentration of CFW or CR containing conditions. The WT versus the *Δste20* strain (A), the WT versus the P_{tcu1}-*sho1* strain (B), the WT versus the P_{tcu1}-*sln1* strain (C) and the WT versus the P_{tcu1}-*ypd1* strain (D). * P<0.05, ** P<0.01, *** P<0.001.

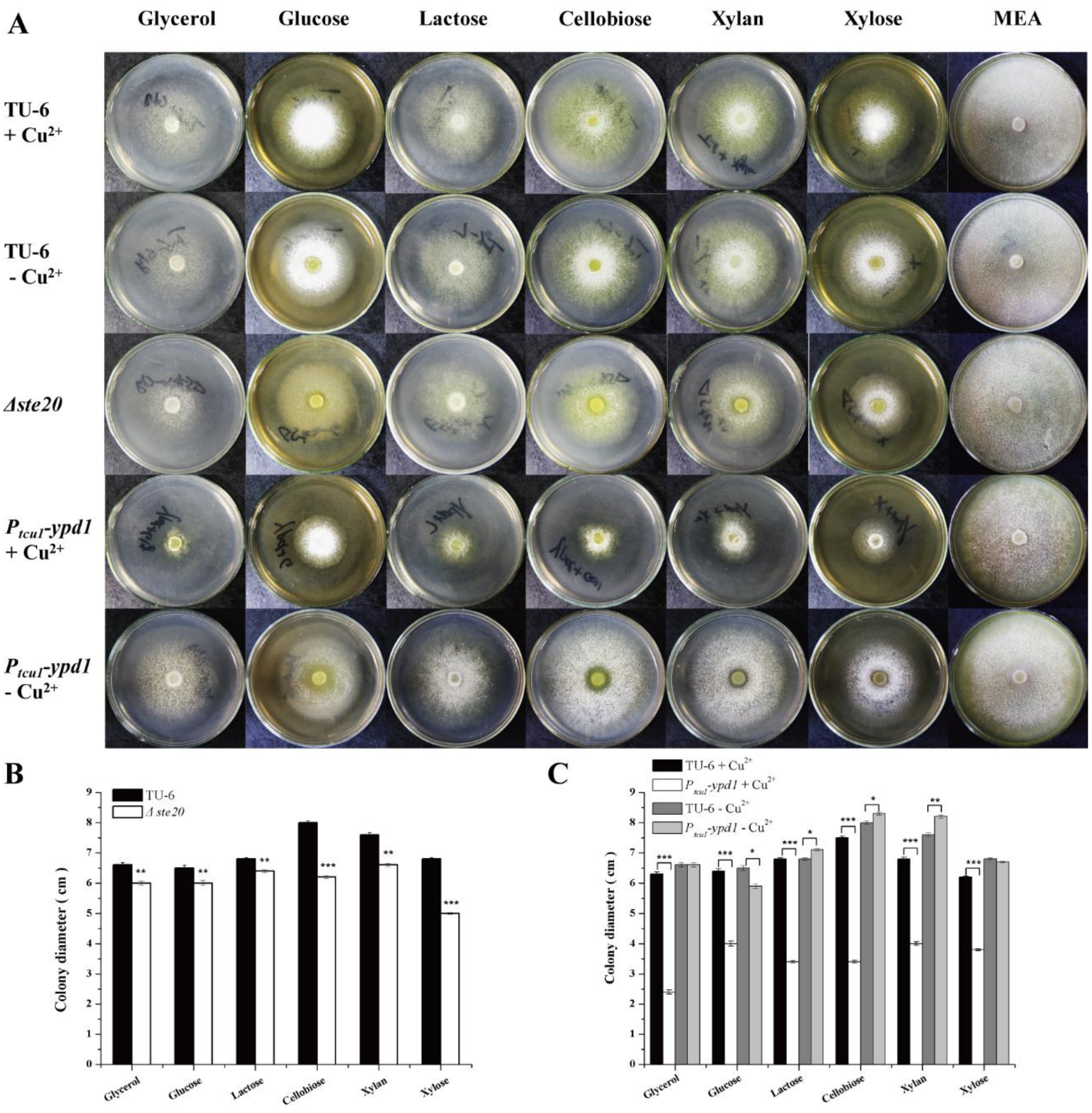

Figure S6. **Characterization of the growth of *Δste20* and P_{tcu1}-*Trypd1* with another transformant.** Another transformant of *Δste20* or P_{tcu1}-*Trypd1* was tested to confirm the results in Figure 3 and Figure 4. (A) Growth of TU-6, *Δste20* and P_{tcu1}-*Trypd1* strains on minimal medium plates containing different carbon sources. Strains were grown at 30 ℃ for 3 days. Three individual replicates of each experiment were performed. (B) Colony diameters of TU-6 and *Δste20* strains on MM plates containing different carbon source. (C) Colony diameters of TU-6 and the P_{tcu1}-*ypd1* strains on MM plates containing different carbon source with or without copper. * $P<0.05$, ** $P<0.01$, *** $P<0.001$.

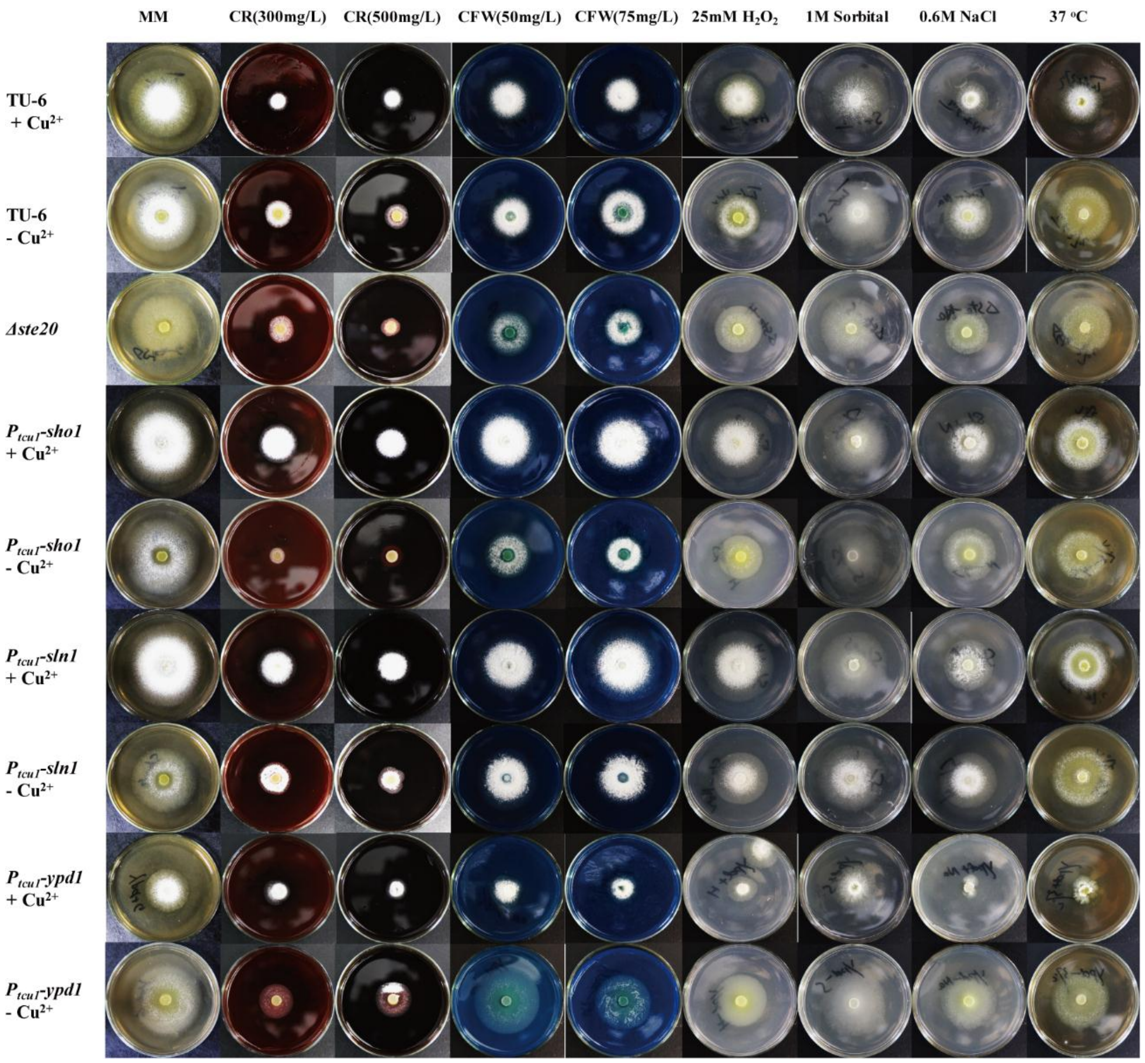

Figure S7. **Stress and cell wall integrity tests of parental and mutant strains using other transformants.** Another transformant of *Δste20*, P_{tcu1}-*Trsho1*, P_{tcu1}-*Trsln1* or P_{tcu1}-*Trypd1* was tested to confirm the results in Figure 5 and Figuire 6. Different concentrations of Calcofluor white (CFW) or Congo red (CR) were added to the minimal medium (MM) plates containing 1% glucose as the sole carbon source. Osmotic stress and oxidative stress tests were performed by adding 25 mM H_2O_2, 1 M sorbital and 0.6 M NaCl to the MM plates, respectively. All the strains were grown at 30 ℃ for 3 days. Thermotolerance was tested by growing all the strains at 37 ℃ for 3 days.
